# Blood triglyceride levels are associated with DNA methylation at the serine metabolism gene *PHGDH*

**DOI:** 10.1038/s41598-017-09552-z

**Published:** 2017-09-11

**Authors:** Vinh Truong, Siying Huang, Jessica Dennis, Mathieu Lemire, Nora Zwingerman, Dylan Aïssi, Irfahan Kassam, Claire Perret, Philip Wells, Pierre-Emmanuel Morange, Michael Wilson, David-Alexandre Trégouët, France Gagnon

**Affiliations:** 1 Division of Epidemiology, Dalla Lana School of Public Health, University of Toronto, Toronto, Canada; 2 Ontario Institute for Cancer Research, Toronto, Canada; 3 Sorbonne Universités, UPMC Univ. Paris 06, INSERM, UMR_S 1166, Team Genomics & Pathophysiology of Cardiovascular Diseases, Paris, France; 4 ICAN Institute for Cardiometabolism and Nutrition, Paris, France; 5 Department of Medicine, Faculty of Medicine, University of Ottawa, Ottawa, Canada; 6 INSERM, UMR_S 1062, Nutrition Obesity and Risk of Thrombosis, Marseille, France; 7 Genetics and Genome Biology Program, SickKids Research Institute, Toronto, Canada

## Abstract

Efficient interventions to reduce blood triglycerides are few; newer and more tolerable intervention targets are needed. Understanding the molecular mechanisms underlying blood triglyceride levels variation is key to identifying new therapies. To explore the role of epigenetic mechanisms on triglyceride levels, a blood methylome scan was conducted in 199 individuals from 5 French-Canadian families ascertained on venous thromboembolism, and findings were replicated in 324 French unrelated patients with venous thromboembolism. Genetic context and functional relevance were investigated. Two DNA methylation sites associated with triglyceride levels were identified. The first one, located in the *ABCG1* gene, was recently reported, whereas the second one, located in the promoter of the *PHGDH* gene, is novel. The *PHGDH* methylation site, cg14476101, was found to be associated with variation in triglyceride levels in a threshold manner: cg14476101 was inversely associated with triglyceride levels only when triglyceride levels were above 1.12 mmol/L (discovery *P*-value = 8.4 × 10^−6^; replication *P*-value = 0.0091). Public databases findings supported a functional role of cg14476101 on *PHGDH* expression. *PHGDH* catalyses the first step in the serine biosynthesis pathway. These findings highlight the role of epigenetic regulation of the *PHGDH* gene in triglyceride metabolism, providing novel insights on putative intervention targets.

## Introduction

Triglycerides are pivotal players in lipid metabolism in health and disease. They are important sources of energy and transporters of dietary fat. In the blood, triglycerides enable the bidirectional transfer of adipose fat and glucose from the liver. Elevated blood triglyceride levels, however, are associated with several chronic conditions. Two large Mendelian randomization studies employing complementary analytic strategies supported a causal role of triglyceride levels in coronary heart disease^1, 2^. Further, elevated triglyceride levels is a key feature in several rare and common metabolic abnormalities, including familial hypercholesterolemia, lipodystrophy, diabetes mellitus types I and II, hypertension, metabolic syndrome and obesity, as well as chronic kidney disease^3–5^. Severe hypertriglyceridemia (fasting levels ≥ 500 mg/dL) is further complicated by the life-threatening complication of acute pancreatitis and its chronic consequences^5^.

Elevated blood triglycerides, defined by fasting levels ≥150 mg/dL (1.7 mmol/L), is present in over 30% of the general adult US population^4^. Thus, elevated blood triglyceride is a common public health challenge with a broad ranging impact on population health. Managing triglyceride levels is of critical importance for patient’s care and overall population health, but it is challenging. Blood triglyceride levels are largely under the influence of body weight and body fat distribution, lifestyle choices, as well as genetic regulation^4^. Robust genetic factors for triglyceride variation have been identified but collectively they account for less than 10% of triglyceride level variability in the general population^6, 7^. On the other hand, over 80% of individuals with high triglyceride levels are either overweight or obese^4^. Lifestyle interventions associated with decreased Body Mass Index (BMI) have shown to reduce blood triglycerides in some contexts (reviewed in^4^) but not all^8^.

Hypertriglyceridemia is undertreated^5^. Pharmacological interventions to reduce blood triglycerides are scarce, and none have been specifically designed to reduce blood triglyceride levels. Fibrates, which are the most efficient and commonly used drugs to reduce triglyceride levels, are associated with 30–50% decrease in triglyceride levels^4^. A large proportion of patients, however, do not reach the therapeutic target^4^. Newer drugs have emerged but their success potential is mitigated by their low tolerability and ineffectiveness in reducing cardiovascular events^9^. Thus, more efficient and tolerable drugs are needed.

Understanding the molecular mechanisms underlying blood triglyceride variation is key to identifying new intervention targets. Epigenetic marks, such as DNA methylation, are molecular mechanisms that are heritable and reversible, and have been associated with a wide range of environmental stimuli and disease phenotypes^10^. Others and we have shown that many blood DNA methylation markers can act as surrogates for DNA methylation marks in effector tissues^11, 12^. Thus, informing function of clinically inaccessible target tissues. This supports the use of DNA methylation profiles in blood as a means to elucidate clinically relevant mechanisms.

DNA methylation marks are highly promising prognostic and therapeutic biomarkers as they are rooted in the mechanisms underlying phenotype expression; thus, more likely to directly guide clinical management of disease^13^. Emerging studies illustrate the potential impact of DNA methylation biomarkers as risk predictors^14, 15^ and intervention targets^16–18^.

Others and we previously reported robust association of blood DNA methylation levels in the *CPT1A* gene with fasting triglyceride levels^12, 19–23^. In addition to methylation levels at *CPT1A* gene, other studies found association of methylation levels at the *ABCG1* gene with triglyceride levels^21–25^ and with hypertriglyceridemic waist phenotype^26^.

To further explore the role of epigenetic mechanisms on fasting triglyceride levels, we conducted a blood methylome study. To reduce environmental and genetic heterogeneity, and increase statistical power, we conducted our discovery analyses in extended French-Canadian families^27^, and replicated our findings in a dataset of unrelated French individuals^28^. To better understand the genetic context and functional relevance of our findings, we investigated proximal genetic variations in the same individuals, as well as *in silico* mRNA expression levels analyses using publicly available datasets. Finally, we explored the role of triglyceride levels in a previously reported association between BMI and methylation levels.

## Materials and Methods

### Study population

#### Overall study design

We applied a multi-design strategy that relied on a family-based design and a set of unrelated individuals, for the discovery stage and replicate stage, respectively. The family design allows for better quality control of the genotypic data and control of population stratification, while the use of a second different design replicates effects without replicating confounders by introducing variation in discovery and replication stages; a recommended approach to tackle such bias^29, 30^.

#### Discovery Study Sample

The French-Canadian Family study on Factor V Leiden (F5L) Thrombophilia (henceforth the F5L family study) included 369 individuals from 5 extended families^27^ of which 255 invited subjects consented to participate in the study. The study was originally designed to investigate genetic determinants of quantitative traits related – or potentially related – to venous thromboembolism, and we recently expanded its scope to include the investigation of epigenetic determinants. The families were identified through the Thrombosis Clinic of the Ottawa Hospital between 2005 and 2006, and probands were ascertained on objectively diagnosed idiopathic venous thromboembolism (i.e. venous thromboembolism in the absence of cancer, myeloproliferative disease, pregnancy, puerperium, prolonged immobilization, trauma, surgery, antiphospholipid syndrome, and inherited thrombophilia) and the F5L mutation. All probands self-reported to be of French-Canadian origin and were free of rare genetic risk factors, including anti-thrombin, protein C, and protein S deficiencies, and homozygosity for the F5L and Factor II G20210A mutations. Participants completed an interviewer-administered questionnaire on their personal and medical information. The research ethics boards of the Ottawa Hospital and the University of Toronto approved this study. An informed consent was obtained for all study participants. All methods were performed in accordance with the relevant guidelines and regulations.

#### Replication Study sample

The MARseille Thrombosis Association (MARTHA) study included 1592 unrelated venous thromboembolism patients of French origin^28^, recruited between January 1994 and October 2005 from the Thrombophilia Center of La Timone Hospital, Marseille, France. The study was designed to investigate venous thromboembolism and quantitative traits related – or potentially related – to venous thromboembolism. Recruitment occurred at least 3 months after the venous thromboembolism event, which was objectively diagnosed by venography, Doppler ultrasound, angiography and/or ventilation/perfusion lung scan. Study subjects were free of chronic conditions, as well as any well-characterized strong genetic risk factors for venous thromboembolism as described above for the F5L family study. Medical and personal histories were obtained from physician interviews. The research ethics boards of “Ministère de la Recherche et de l’Innovation” approved this study. An informed consent was obtained for all study participants for the purposes of data analysis and publications. All methods were performed in accordance with the relevant guidelines and regulations.

### Measurements

#### DNA methylation

In both the F5L family and MARTHA studies, DNA was extracted from peripheral blood using a salting out procedure adapted from^31^. Bisulphite conversion and DNA methylation measurements were performed at The Center for Applied Genomics, Toronto, Canada in 227 subjects from the F5L family study and in 350 subjects from the MARTHA study randomly selected from those with whole blood DNA available^12^. Eleven duplicate samples from both studies were included for quality control. The DNA samples from the F5L family and MARTHA studies were mixed randomly by plate and batch to minimize potential technical biases between the study samples. Bisulphite conversion was performed on 1 µg genomic DNA for each sample using the Qiagen EpiTect 96 Bisulphite Kit and 200 ng of bisulphite-converted DNA at 50 ng/µl was independently amplified, labelled and hybridized to Infinium HumanMethylation450 Bead Chip microarrays. For each sample, the intensities of the methylated and unmethylated signals were measured at 485,577 CpG sites using the Illumina iScan (with default settings). DNA methylation refers to the addition of a methyl group on a CpG site. At a single molecule level, methylation is a then binary mark, i.e. either methylated or unmethylated^32^ However, the microarray measurements at the tissue level (e.g. blood) reflect the average over all copies of DNA present in the tested sample. As a consequence, DNA methylation measurement at any CpG site is a quantitative signal that can be viewed as the percentage of cells methylated in the sample. Methylation levels at individual CpG sites were reported as β-value, i.e. the ratio of the methylated probe intensity to the overall intensity (sum of methylated and unmethylated probe intensities).

Methylation data from the F5L family and MARTHA studies were merged for quality control and data normalization as previously described^11, 33^. The data were normalized using SWAN^34^, Noob^35^ and a dye bias adjustment method proposed by Illumina. Individual outliers were detected based on a principal component analysis, and four individuals from the F5L family study were excluded. Probe quality was assessed by the detection *P*-value as defined in the *minfi* R package, and low detection *P*-values indicated that the probe signal differed from the background signal. We excluded 6033 probes with detection *P*-value > 0.05 in more than 5% of the samples, as well as 66,877 polymorphic and 30,969 cross-reactive probes identified by Chen *et al*.^36^. The final dataset included 378,594 autosomal probes.

#### Genotyping

Measurement and procedures for quality control of the genotypic data are described in the Supplemental Data. Briefly, genome-wide genotypes in the F5L family study were typed using the Illumina 660W-Quad Beadchip. SNPs with call rate ≤90% and minor allele count <20 were excluded^28^. Family structures were validated using genome-wide microsatellite markers and SNP data as described elsewhere^27^.

MARTHA subjects were genotyped using the Illumina 610 or 660W-Quad Beadchips^28^. SNPs with a Hardy-Weinberg equilibrium *P*-value < 10^−5^, a call rate <99% or a minor allele frequency <1% were excluded from the final dataset.

#### Lipid-related measures and BMI

Blood samples were collected from study participants after a 12 hour overnight fast, and F5L family study participants had additionally been instructed to abstain from smoking in the previous 12 hours. In the F5L family study, lipoproteins were measured at the Lipid Research Laboratory at St-Michael’s Hospital as described elsewhere^37^. Blood samples were collected from MARTHA participants after a 12 hours overnight fast at the Department of Haematology of La Timone Hospital. In both studies, serum levels of triglycerides (in mmol/L), very low-density lipoprotein (VLDL, in g/L) and high-density lipoprotein (HDL, in g/L) were assessed by spectrophotometry. The low-density lipoprotein (LDL, in g/L) level was calculated with the Friedewald equation. Triglyceride levels were measured in 239 F5L family study subjects and in 327 subjects of the 350 epigenotyped MARTHA subjects. Subjects self-reported their height and weight, and BMI was calculated as body mass divided by the square of body height.

#### Cell type proportions

The percentage of cells methylated may vary depending on the tissue - and cell types that composed that tissue - in which it is measured. In the case of whole blood, the DNA methylation measured is that of all leukocyte subtypes together (e.g. neutrophils, monocytes, etc.). Thus, the percentage of cells methylated (i.e. methylation level) in whole blood is an aggregate measure of the proportion from each blood cell types. Inter-individual differences in blood cell type proportions can thus affect blood DNA methylation levels^38^, and should be accounted for in the analyses. In the MARTHA study, the proportions of lymphocytes, monocytes, neutrophils, eosinophils and basophils were directly measured from whole blood using the ADVIA 120 Haematology System (Siemens Healthcare Diagnostics, Deerfield, IL). Cell type proportions were not measured in the F5L family study. Instead, we used Remove Unwanted Variation (RUV) method^39, 40^ to capture the cell type proportions as described in Supplemental Data.

### Statistical analysis

Due to a highly right-skewed distribution, fasting triglyceride levels were log transformed to improve model fit. DNA methylation β-values were logit transformed into M-values as recommended by Du *et al*.^41^.

#### Methylome association scan

A methylome association scan of triglyceride levels was conducted. A variance components model was fit in the F5L family study to account for familial correlation. Factor components from the Remove Unwanted Variation (RUV) method^39, 40^ were included in the model to capture cell type proportions and batch effects, and the model was additionally adjusted for age (years) and sex. Triglyceride level was treated as independent variable, and methylation level at each CpG site (M-value) was regressed on triglycerides and potential confounding factors. The statistical significance of the association of triglycerides with DNA methylation levels was tested using the likelihood ratio test. Full details of the statistical methods are provided in the Supplemental Data.

CpG sites that met the genome-wide significance threshold of FDR *q*-value ≤ 0.05 were further tested for association with triglyceride levels in the MARTHA study. Models were adjusted for age, sex and proportions of measured lymphocytes, monocytes, basophils and eosinophils. *P*-values ≤ 0.05 after Holm-Bonferroni correction^42^ were considered statistically significant.

#### Technical validation and Sensitivity analyses

To assess the robustness of our novel findings, technical validation and sensitivity analyses were performed. Details are provided in the Supplementary materials. Briefly, technical validation of the novel DNA methylation findings was performed by qRT-PCR, and various models to account for cellular heterogeneity and other potential confounders were tested and compared in both studies. Approaches to account for cellular heterogeneity include the Remove Unwanted Variation (RUV) method^39, 40^ components and adjustments for the estimated or the true cell type proportions. The influence of additional adjustments for potential confounders (e.g. lipid lowering medication, oral contraceptive use and current smoking) was also examined. Finally, we used the gaphunting approach^43^ to assess whether the CpG sites showed a clustered distribution.

#### Follow-up analyses

For the novel identified triglyceride-DNA methylation locus, a difference in the strength of association between our discovery and replication studies was observed. To understand the source of this variability, further in-depth analyses were conducted. First, a non-linear association between methylation and triglyceride levels was explored. A Generalized Additive Model (GAM)^44^ with same covariates adjustment as in the discovery model was fitted. In the F5L family study, the family clusters were treated as random effect to adjust for inter-individual correlation. Upon confirmation of the non-linear association, to quantify the associations, further analyses were conducted using piecewise linear regression models with a pre-determined breakpoint value. The piecewise regression model approach assumes linear relationship within each defined segment on the independent variable’s scale, and estimates linear associations within defined intervals separately^45^. Full details of the method are described in the Supplemental Data.

A recent methylome study showed an inverse association between BMI and DNA methylation levels^46^. Since triglycerides and BMI are positively correlated, additional analyses to clarify the interrelationship among BMI, triglycerides and DNA methylation levels at the discovered locus were conducted. First, the strength of the association between BMI and methylation levels at the locus was estimated applying a linear regression model adjusted for same set of covariates as described above. Then, triglyceride level was added as a covariate in the model. The regression coefficient estimates of BMI between these two models were compared. A reduction in the magnitude of the coefficient toward the null when adjusting for triglyceride levels suggests that triglyceride levels lay in the pathway linking BMI to methylation levels. Similarly, the BMI-triglyceride-methylation relationship was investigated by comparing models for triglycerides–methylation association, with or without adjustment for BMI. The direct effect of BMI on methylation levels was also estimated using Structural Equation Modelling (SEM). Triglyceride levels were assumed to lie in the pathway linking BMI and methylation levels. The models were adjusted for age, sex and cell type composition in both studies.

#### Genetic association analysis

**Figure 1:**
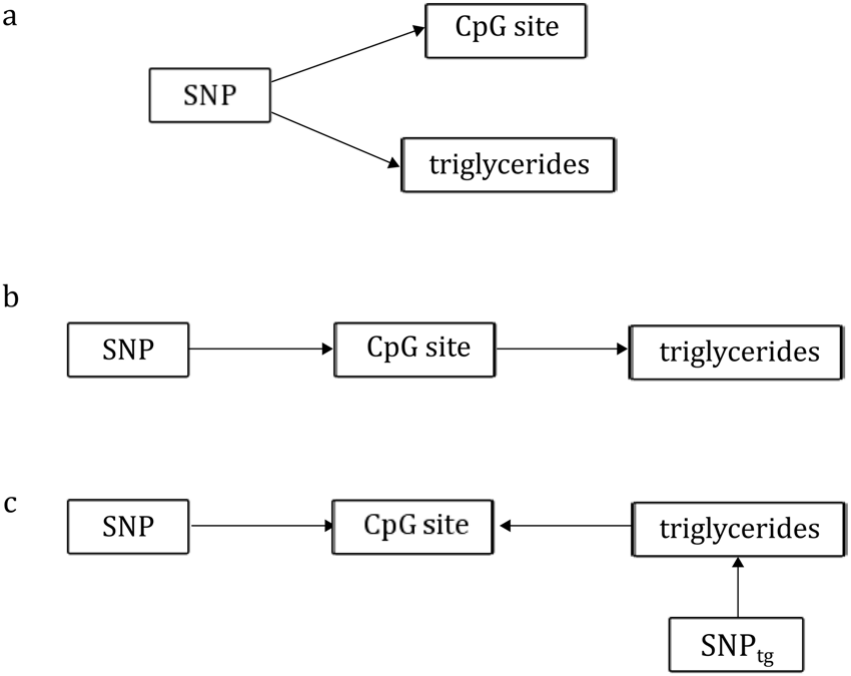
Possible relationships between a SNP, a CpG site and triglyceride levels. The SNP is assumed to act on the methylation levels at the CpG site: (**a**) The SNP can cause variation independently in the level of methylation and triglycerides (common cause); (**b**) The CpG site can be on the pathway between the SNP and triglyceride levels. In this case, the SNP is expected to be associated with triglyceride levels; (**c**) The methylation change can be the consequence of the triglyceride levels variation (reverse causation). Reverse causation can be inferred with a genetic variant (SNPtg) associated with triglyceride levels.

Genetic analysis was carried out to assess whether the observed associations between methylation and triglyceride levels were due to underlying common SNPs^33, 47^ (Fig. 1a). SNPs within 1 Mb of the CpG sites were first tested for association with methylation levels in the F5L family study, and those with FDR *q*-value ≤ 0.05 were tested for validation in the MARTHA study. SNPs that were associated with DNA methylation in the MARTHA study after Holm-Bonferroni correction were then tested for association with triglyceride levels across both study samples. These SNP-triglycerides associations were further investigated *in silico* in the *Global Lipids Genetics Consortium* meta-analysis results, which involved more than 90,000 individuals^48^. Finally, the identified SNPs were used as instrumental variables in a Mendelian randomization framework^49, 50^ to examine a putative causal effect of DNA methylation at the CpG site on triglyceride levels (Fig. 1b). The possibility of reverse causation (Fig. 1c) was examined using the 32 SNPs reportedly associated with triglyceride levels, individually and as a genetic score as described in the Supplemental Data.

All analyses were conducted using R version 3.0.1. Variance components models were fitted with the *pedigreemm* package, GAM models with the *mgcv* package, and SEM models with the Lavaan package^51^.

## Results

### Study population characteristics

**Figure 2:**
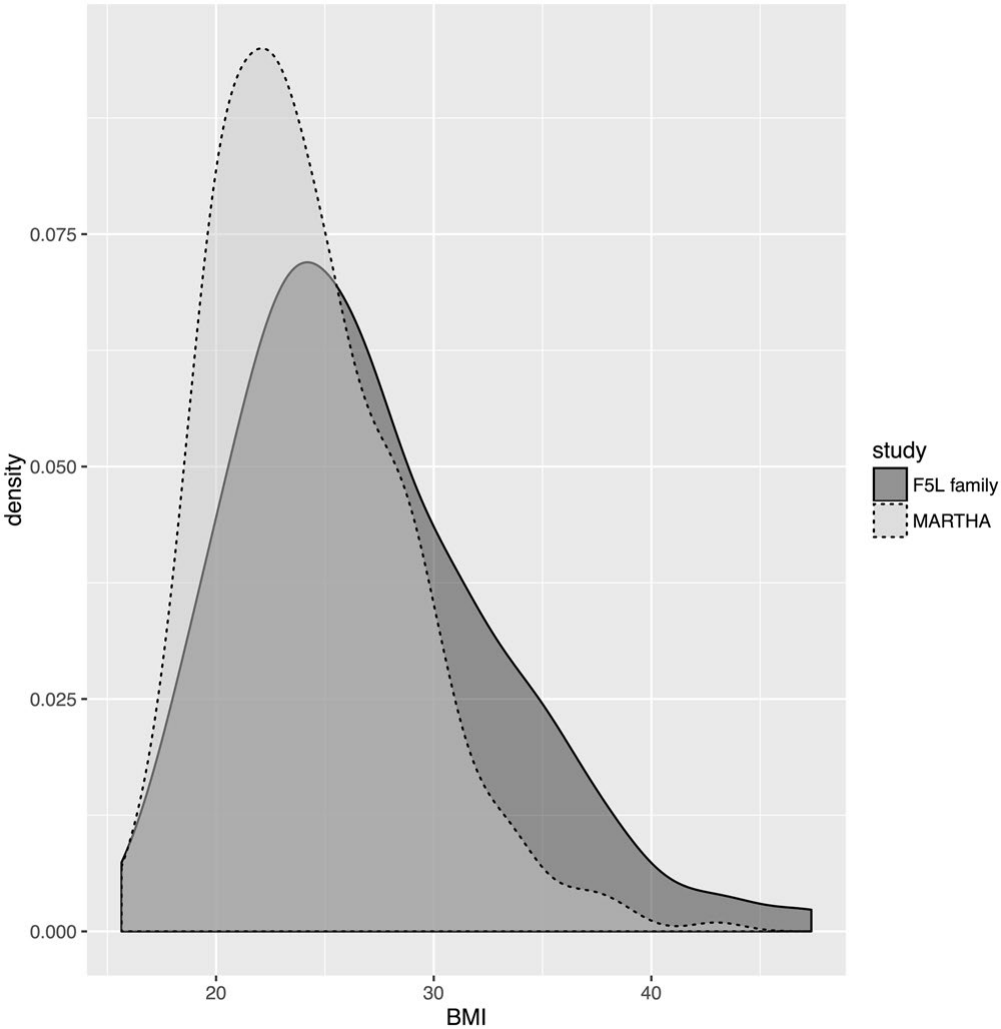
Density plot of triglyceride levels in the F5L family and MARTHA studies.

Data on triglyceride levels, genotypes, and DNA methylation levels were available from 199 individuals in the F5L family study and from 324 individuals in MARTHA (Table 1). The two study populations differed in a few aspects: compared to the MARTHA study participants, who are all venous thromboembolism patients, only 11 participants in the F5L family study had a venous thromboembolism. The F5L family participants had, on average, a higher BMI (26.8 kg/m^2^ vs 24.2 kg/m^2^; *P*-value = 1.6 × 10^−4^) and higher triglyceride levels (1.5 mmol/L vs 1.0 mmol/L; *P*-value = 9.7 × 10^−10^); and were on average 4 years younger. Triglyceride levels in the F5L family study were systematically shifted toward higher values compared to the levels observed in the MARTHA study (Fig. 2). The MARTHA study was mainly composed of females (77.5%), whereas the F5L family study had an equal proportion of males and females. Participants in the two studies were similar with respect to LDL and HDL levels. Lipid-lowering drug use was low in both studies (7% and 11%; *P*-value = 0.15).

**Table 1:** Subject characteristics in the F5L family and MARTHA studies.

	F5L family study Discovery dataset Mean ± SD or N (%) N = 199	MARTHA study Replication dataset Mean ± SD or N (%) N = 324
Males*	93 (46.7%)	73 (22.5%)
Age (year)*	39.6 ± 16.9	44.1 ± 14.3
FVL heterozygote	48 (24%)	89 (28%)
VT prevalence*	11 (5.5%)	324 (100%)
BMI (kg/m^2^)*	26.8 ± 6.1	24.2 ± 4.4
Current Smoker	50 (25.5%)	94 (29%)
Lipid-lowering drug use	12 (6.8%)	33 (10%)
Anticoagulant use	4 (2.3%)	0 (0%)
Triglycerides (mmol/L)*	1.5 ± 0.9	1.0 ± 0.5
VLDL (g/L)	0.4 ± 0.4	NA
LDL (g/L)	3.1 ± 0.9	3.3 ± 0.9
HDL (g/L)	1.4 ± 0.4	1.5 ± 0.4

*P-value < 0.01 from the statistical test of equality of the trait distribution of the two datasets; A Kolmogorov-Smirnov test was used for continuous traits and a Fisher exact test for binary traits.

### Methylome association scan

The methylome-wide association scan of triglyceride levels in the discovery data set (i.e. the F5L family study) identified two CpG sites reaching genome-wide significance level. These results are shown in Manhattan plot of the Supplementary Fig. S1; the associated Q-Q plot is shown in Supplementary Fig. S2. DNA methylation levels at these two CpG sites (cg06500161 and cg14476101) were found associated with triglyceride levels in the F5L family study and replicated in the MARTHA study (Table 2). The first CpG site, cg06500161, is located in the *ABCG1* gene. DNA methylation at this site was recently reported to be positively associated with triglyceride levels in a population-based cohort^25^ and in female patients with familial hypercholesterolemia^24^. We detected a similar strength of the association with respect to magnitude and sign in the MARTHA study (β = 0.11, *P*-value = 1.92 × 10^−8^) as in the F5L family study (β = 0.11, *P*-value = 1.5 × 10^−7^). The second locus is novel, and maps to the *PHGDH* gene. DNA methylation at cg14476101 was inversely associated with triglyceride levels in the F5L family study (β = −0.21, *P*-value = 2.3 × 10^−7^, 95% confidence interval (CI) (−0.29, −0.13)) and in the MARTHA study (β = −0.08, *P*-value = 4.8 × 10^−2^, 95% CI (−0.16, −6.7 × 10^−3^). Variation in triglyceride levels explained 13% and 6.4% of the methylation level variance at this CpG in the F5L family study and in the MARTHA study, respectively. The effect of triglyceride levels on DNA methylation was independent of the effects of age, sex and cell type proportions. Additional adjustments for lipid lowering medications, oral contraceptive use or current smoking did not alter the association of triglyceride levels with methylation levels at cg14476101 (Supplementary Table S1). In addition, cg14476101 is not annotated as SNP probe in the Illumina provided manifest. Based on the concordance between whole-genome bisulphite sequencing data and the Illumina assay^52^, the signal provided by cg14476101 is not affected by a SNP. In addition, the cg14476101 did not show clustered distribution in either study. Technical validation of cg14476101 by bisulfite qRT-PCR compared to H450M quantification showed high correlation, 0.865 (Supplementary materials Supplementary Fig. S11).

**Table 2:** Statistically significant associations between triglyceride and methylation levels in the F5L family study and in the MARTHA study.

CpG site	Position (Gene)	F5L family study		MARTHA study	
		β (95% CI)	P-value	β (95% CI)	P-value
cg06500161	21: 43,656,587 (*ABCG1*)	0.11 (0.073, 0.15)	1.5 × 10^−7^	0.11 (0.075, 0.15)	1.92 × 10^−8^
cg14476101	1: 120,255,992 (*PHGDH*)	−0.21(−0.29, −0.13)	2.3 × 10^−7^	−0.082 (−0.16, −6.7 × 10^−3^)	0.048

Associations were tested using a linear regression model (a variance components model in the F5L family study) where cg14476101 methylation levels expressed as M-value were analysed as the outcome, and triglyceride levels as a predictor. Models were adjusted for sex, age and cell type proportions (RUV components in the F5L family study). Statistical significance was assessed with a FDR in the F5L family study (q-value ≤ 0.05) and with a Holm-Bonferroni correction in the MARTHA study (p_corrected ≤ 0.05). CI, confidence interval.

Hereafter, the focus for the remaining analyses is on the novel cg14476101 *PHGDH* CpG site. A regional plot of the association of triglyceride levels with DNA methylation levels in the vicinity of cg14476101 is shown in Supplementary Fig. S3.

### Non-linear triglyceride-CpG relationship

**Figure 3:**
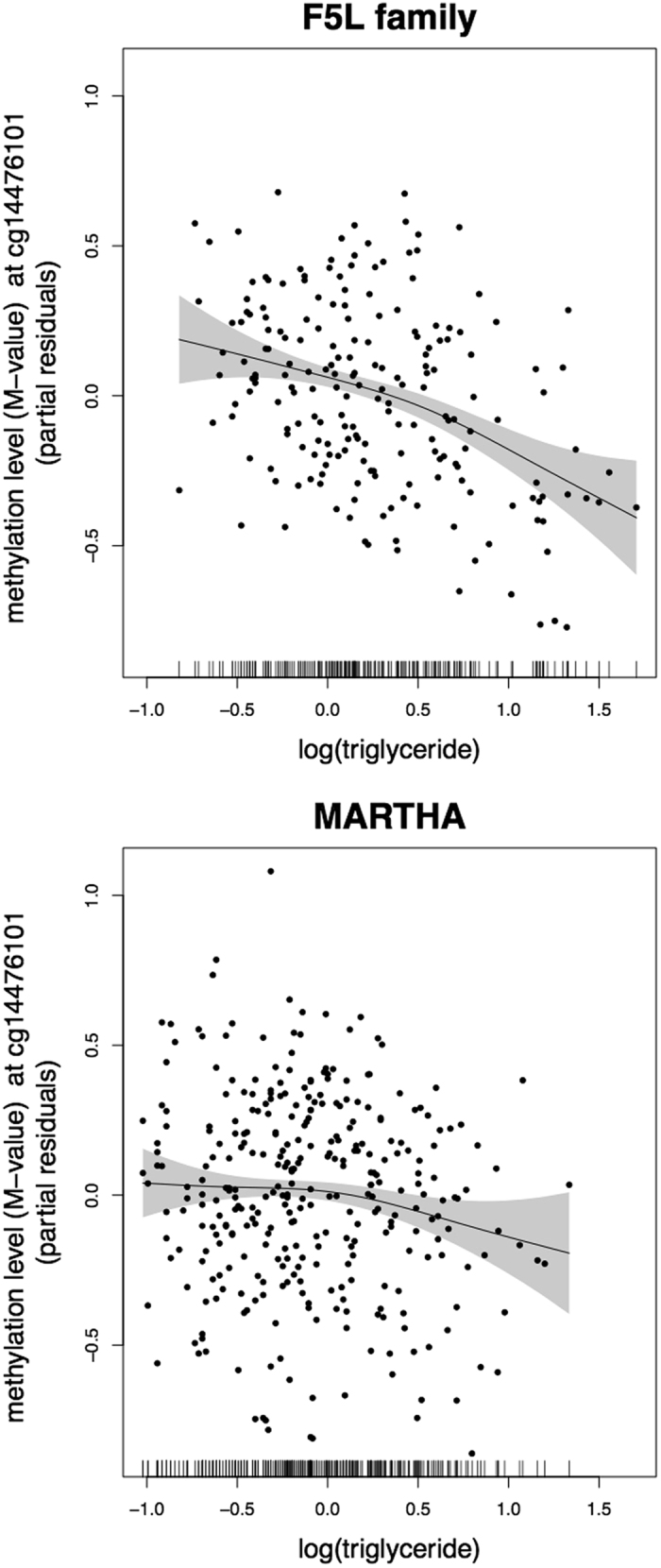
Partial residual plots from the GAM model showing the relationship between triglyceride and the *PHGDH* cg14476101 methylation levels in the F5L family and MARTHA studies. The non-linear relationship was modelled with a GAM model adjusted on age, sex and cell type proportions (RUV component in the F5L family study). A random effect was added to the model for the analysis of the F5L family study data to adjust for the relatedness among the family members.

Although the association between methylation levels at *PHGDH* cg14476101 and triglyceride levels was replicated in MARTHA, the strength of the association was slightly different across both samples. To investigate the underlying cause of this disparity, a non-linear relationship between methylation and triglyceride levels that may not be captured by a linear model was considered. Under the generalized additive model (GAM) framework, the estimated degrees of freedom for triglyceride levels were similar in both studies: 1.91 (*P*-value = 1.3 × 10^−6^) in the F5L family study and 1.97 (*P*-value = 0.085) in the MARTHA study. These results suggest a non-linear association between triglycerides and methylation levels. A visual inspection of the GAM plots showing the cg14476101 methylation-triglyceride levels relationship (Fig. 3) indicated a similar trend in both studies. A monotonic inverse association between methylation and triglyceride levels was observed. Methylation levels rapidly decreased when triglyceride levels were above a threshold of approximately 1.1 mmol/L in the MARTHA study and 1.6 mmol/L in the F5L family study. This trend was more apparent in the F5L family study. The different threshold and slope observed between these two study samples likely reflect the difference in their triglyceride level distributions observed between the two studies: the distribution was shifted to the left in the MARTHA study compared to the F5L study. Estimation of the non-linear effects separately in each study showed wider confidence intervals at higher values in the MARTHA study compared to confidence intervals at lower values; and confidence intervals in the lower range of the distribution in the F5L family were larger than those observed in the MARTHA study (Fig. 3). Consequently, the strength of associations was estimated with higher uncertainty for lower triglyceride levels in the F5L family study compared to the MARTHA study; and in the MARTHA study, the level of uncertainty was higher in the higher value range of the distribution. That may impact the point estimate of the threshold in both studies.

To compare and quantify the observed threshold effect, a piecewise linear regression with a triglyceride levels breakpoint at 1.12 mmol/L was fitted (Table 3). This breakpoint value was selected based on the American Heart Association guidelines for triglyceride levels management^4^, and aligned with the observed thresholds in this study. Triglyceride levels below the breakpoint were not statistically associated with methylation levels in either dataset, while triglyceride levels above the breakpoint were statistically significant and of similar strength in both the F5L family study (β = −0.26, *P*-value = 8.4 × 10^−6^, 95% CI (−0.37, −0.14)) and the MARTHA study (β = −0.25, *P*-value = 9.1 × 10^−3^, 95% CI (−0.44, −0.062)).

**Table 3:** Association of methylation levels at *PHGDH* cg14476101 with triglyceride levels by strata in the F5L and MARTHA studies.

	F5L family study			MARTHA study		
	N	β (95% CI)	P-value	N	β (95% CI)	P-value
triglyceride levels ≥1.12 mmol/L	113	−0.26 (−0.37, −0.14)	8.4 × 10^−6^	102	−0.25 (−0.44, −0.062)	0.0091
triglyceride levels <1.12 mmol/L	86	−0.12 (−0.31, 0.081)	0.24	222	0.022 (−0.11, 0.15)	0.74

Associations were tested using a linear regression model (a variance components model in the F5L family study) where cg14476101 methylation levels expressed as M-value were analysed as the outcome, and triglyceride levels as a predictor. We estimated the effects by triglyceride levels strata (triglyceride levels ≥1.12 mmol/L and triglyceride levels <1.12 mmol/L) using a piecewise linear regression model. Models were adjusted for age, sex and cell type proportion (RUV component in the F5L family study). CI, confidence interval; N, number of individuals in the stratum.

### Genetic association analysis

Of the 217 genotyped SNPs within 1 Mb of cg14476101, six were associated with methylation levels at cg14476101 in both the F5L family and MARTHA studies (Supplementary Table S2). However, none of these SNPs were associated with triglyceride levels in either dataset, nor were they significant in the *Global Lipids Genetics Consortium* meta-analysis dataset (Supplementary Table S3). Thus, the relationship between cg14476101 methylation and triglyceride levels appears to be independent of proximal genetic variants. These results can be interpreted within a Mendelian Randomization framework. The six genetic variants were assumed to influence methylation levels at cg14476101, and the absence of association between these SNPs and triglyceride levels indicates that variation in cg14476101 methylation levels is unlikely a causal factor in triglyceride level variation. On the other hand, the triglyceride-associated genetic score showed consistent effect size and direction across datasets with cg14476101 methylation levels (β = −0.089; *P*-value = 0.48 in the F5L family study and β = −0.13; P-value = 0.11 in the MARTHA study, Supplementary Table S4). Thus, these results are consistent with reverse causation but are inconclusive.

### Triglycerides-CpG relationship with BMI

An association between *PHGDH* cg14476101 methylation levels and BMI was recently reported^46^. Given the well-established relationship between triglycerides and BMI, the potential role of triglyceride levels in the reported BMI-cg14476101 association was investigated (as described in Supplementary Fig. S5).

First, the effect of BMI on cg14476101 methylation levels was estimated. The association was marginal within the F5L family (β = −7.1 × 10^−3^, *P*-value = 0.040) and MARTHA (β = −6.9 × 10^−3^, *P*-value = 0.11) studies as compared to the one observed for triglycerides. However, the strengths of the associations were similar and directionally concordant with those previously reported^46^. The strengths of the association of BMI on cg14476101 methylation levels were then compared using a linear model with and without triglycerides as a covariate. BMI was not associated with methylation levels in either study (Table 4). In addition, adjusting for triglyceride levels in the model reduced the effect of BMI on cg14476101 methylation levels by 54% and 29%, respectively, in the F5L family study and in the MARTHA study. These results suggest that triglyceride levels variation likely plays an intermediate role in the BMI-cg14476101 relationship. Conversely, BMI did not significantly attenuate the association between cg14476101 methylation and triglyceride levels in piecewise linear models (3.1% and 3.6% effect estimate reduction, respectively, in the F5L family and MARTHA studies), suggesting that BMI is neither a confounder nor a mediator in the DNA methylation-triglyceride levels relationship (Supplementary Fig. S5; Table 5). We further conducted mediation analyses using structural equation modelling in the F5L and MARTHA datasets assuming the pathway to be: BMI → triglyceride levels → methylation levels (Supplementary Fig. S6). Results from both datasets indicate that the BMI effect on methylation level at the cg14476101 CpG site were mediated through the triglyceride levels (Table 6).

**Table 4:** Effect of BMI on methylation levels at *PHGDH* cg14476101 without and with adjustment for triglyceride levels in the F5L family and MARTHA studies.

	F5L family study	MARTHA study
Effect size of BMI without triglyceride levels adjustment	−0.011	−0.0069
95% CI	(−0.014, −2.4 × 10^−3^)	(−0.015, 1.6 × 10^−3^)
*P*-value	0.04	0.11
Effect size of BMI with triglyceride levels adjustment	−3.3 × 10^−3^	−4.9 × 10^−3^
95% CI	(−0.010, 4.0 × 10^−3^)	(−0.015, 4.8 × 10^−3^)
*P*-value	0.36	0.33

Associations were tested using a linear regression model (a variance components model in the F5L family study) where cg14476101 methylation levels expressed as M-value were analysed as the outcome, and BMI as a predictor. Models were adjusted for age, sex and cell type proportion, with and without triglyceride levels. CI, confidence interval.

**Table 5:** Effect of triglyceride levels on methylation levels at *PHGDH* cg14476101 by strata without and with adjustment for BMI in the F5L family and MARTHA studies.

	Direct effect, β_BC_ Coef (se), P-value	Indirect effect, β_BT_ * β_TC_ Coef (se), P-value	Total effect β_BC_ + β_BT_ × β_TC_ Coef (se), P-value	Goodness of fit RMSEA (LI, UI)
F5L Family study	−0.12 (0.11), 0.26	−0.195 (0.05), <0.001	−0.32 (0.106), 0.003	<0.001 (<0.001, 0.092)
MARTHA study	−0.14 (0.12), 0.26	−0.081 (0.05), 0.098	−0.22 (0.112), 0.051	<0.001 (<0.001, 0.063)

Associations were tested using a linear regression model (a variance components model in the F5L family study) where cg14476101 methylation levels expressed as M-value were analysed as the outcome, and triglyceride levels as a predictor. We estimated the effects by triglyceride levels strata (triglyceride levels ≥1.12 mmol/L and triglyceride levels <1.12 mmol/L) using a piecewise linear regression model. Models were adjusted for age, sex and cell type proportion (RUV component in the F5L family study) with and without BMI. CI, confidence interval; N, number of individuals in the stratum.

**Table 6:** Mediation analysis using a structural equation modeling.

	F5L family study		MARTHA study	
	triglycerides <1.12 mmol/L N = 86	triglycerides ≥1.12 mmol/L N = 113	triglycerides <1.12 mmol/L N = 222	triglycerides ≥1.12 mmol/L N = 102
Effect size of triglyceride levels without BMI adjustment	−0.12	−0.26	0.022	−0.25
95% CI	(−0.31, 0.081)	(−0.37, −0.14)	(−0.11, 0.15)	(−0.44, −0.062)
P-value	0.24	8.4 × 10^−6^	0.74	0.0091
Effect size of triglyceride levels with BMI adjustment	−0.097	−0.25	0.038	−0.24
95% CI	(−0.30, 0.11)	(−0.36, −0.13)	(−0.099, 0.17)	(−0.42, −0.046)
P-value	0.34	2.4 × 10^−5^	0.59	0.036

Mediation analyses were performed using structural equation modelling in both F5L family and MARTHA studies assuming the pathway to be: BMI (B) → triglyceride levels (T) → methylation levels (C). Models were adjusted for age and sex and cell type proportion accordingly in each dataset. RMSEA <0.08 in general shows good fit. RMSEA, root mean square approximation. The non-linear relationship was modelled with a GAM model adjusted on age, sex and cell type proportions (RUV component in the F5L family study). A random effect was added to the model for the analysis of the F5L family study data to adjust for the relatedness among the family members.

### DNA methylation in the *ABCG1* and *CPT1A* genes

As previously reported, associations of methylation levels at the *ABCG1* (cg06500161) and *CPT1A* (cg00574958) genes with triglyceride levels were observed (Supplementary Table S5). Thus, providing external validity to our findings, and highlighting the value of our small but well-characterized study samples for epigenomic investigations. To assess whether non-linearity was also a characteristic of the *CPT1A* and *ABCG1* DNA methylation-triglyceride relationships, a GAM model was applied. None of the associations showed a non-linear trend, and results confirmed the linear relationship (*P*-value = 4.5 × 10^−6^ with a degree of freedom estimated at 1 for cg06500161; *P*-value = 4.0 × 10^−4^ with a degree of freedom estimated at 1 for cg00574958). Finally, to assess whether methylation levels at the triglyceride-associated *PHGDH* CpG site were influenced by methylation levels at the *ABCG1* and *CPT1A* sites, additional models adjusting for these sites were tested. Adjustments for methylation levels at the *ABCG1* and *CPT1A* CpG sites did not affect the association between triglyceride levels and the *PHGDH* CpG site, suggesting that their effects are independent (Supplementary Table S6).

### Gene expression association

The cg14476101 probe tags a CpG site located in the first intron of the *PHGDH* gene on chromosome 1. In order to assess the potential functional relevance of the *PHGDH* gene and triglyceride levels variation, we looked at the association of the *PHGDH* expression with the methylation levels at the *PHGDH* CpG site and with the triglyceride levels. Since gene expression levels were not measured in F5L family and MARTHA studies, we turned to two public datasets: eMS^53^ and GHS Express^54^. eMS is a database of associations between DNA methylation levels and gene expression levels in purified peripheral monocytes from the Multi-Ethnic Study of Atherosclerosis^55^. GHS Express is a public database of expression quantitative trait loci, which includes results of associations between triglyceride levels and monocyte gene expression levels. In the eMS dataset, increased methylation levels at cg14476101 were significantly associated with lower *PHGDH* expression (β = −0.42, *P*-value = 2.2 × 10^−41^, N = 1264). In the GHS Express dataset, the expression of *PHGDH* was positively associated with triglyceride levels (β = 0.19, *P*-value = 1.5 × 10^−9^, N = 1490). Taken together, these results support the association between triglyceride and methylation levels at cg14476101, and suggest a novel role of the *PHGDH* gene in triglyceride metabolism. For comparison, gene expressions of *CPT1A* and *ABCG1* were also positively associated with triglyceride levels in the GHS Express dataset (β = 0.036, *P*-value = 5.4 × 10^−3^ and β = −0.11, *P*-value = 2.1 × 10^−13^, respectively). Methylation levels in the *CPT1A* and *ABCG1* genes (cg00574958 and cg06500161) were not available in the eMS dataset.

## Discussion

Applying a multi-design strategy and two data sets that have previously been shown to be efficient for the discovery and replication of genetic and epigenetic variants associated with cardiovascular traits, this work reports associations between fasting triglyceride and blood methylation levels at two CpG sites in two genes: *PHGDH* and *ABCG1*. Although both datasets used in this study have a common underlying ascertainment scheme favouring the investigation of venous thrombosis, our DNA methylation-triglyceride associations are likely generalizability to other populations, as previously shown in Gagnon *et al*. 2014.

DNA methylation levels at *PHGDH* cg14476101 were inversely associated with blood triglycerides. Mendelian randomization and genetic score analysis findings were consistent with triglyceride levels most likely affecting DNA methylation at these sites, rather than vice-versa. In addition, the observed association between DNA methylation at the *ABCG1* cg06500161 and blood triglycerides provided robustness to the above novel findings as the cg06500161-triglycerides association had previously been reported^24, 25^.

The cg14476101 site is located in the first intron of the *PHGDH* gene. According to the Roadmap Epigenome browser^56^, the site is located in the liver in a region enriched for the posttranslational histone modification H3K27Ac (acetylation of lysine 27 of the H3 histone protein) that is indicative of active enhancers and in H3K4me3 (Histone 3 lysine 4 trimethylation) a modification that is indicative of proximal promoter regions (Supplementary Fig. S7). In the peripheral blood mononuclear cells, the CpG site lies in close proximity to a region enriched in H3K4me4. These results suggest that the *PHGDH* CpG site is located in or close to a promoter region. Methylation in a promoter region is often associated with repression of gene expression^57^. This is consistent with the observed expression results in two public datasets: methylation levels at cg14476106 were inversely associated with *PHGDH* expression. In addition, an inverse association between the expression of *PHGDH* and fasting triglycerides was observed in circulating monocytes. The results show that the *PHGDH* gene is likely involved in triglyceride metabolism. But our analysis is limited to the monocytes. Further studies are needed to understand the relationship between the methylation levels at cg14476101, the expression of *PHGDH* and fasting triglycerides and in different blood cell types.

While results from the GHS Express and eMS indicated that *PHGDH* is expressed in monocytes, the Roadmap Epigenome browser showed that *PHGDH* was barely expressed or completely repressed in peripheral blood mononuclear cells (Supplementary Fig. S7). This disparity may be due to the fact that monocytes only make up 2 to 8% of white blood cells^58^. Thus, the effect on monocytes might be mitigated by other white blood cell types that have low or no expression of *PHGDH*. Under the ChromHMM prediction model^59^, the region that contained cg14476101 is an active transcription starting site and is close to a weak transcription region. These lines of evidence indicate that, under normal conditions, *PHGDH* is expressed in monocytes and is less likely to be actively transcribed. This is consistent with the study findings: only after reaching a clinically relevant triglyceride levels threshold did methylation levels decrease. This implies that when triglyceride levels are below the threshold, *PHGDH* is less likely to be expressed; above the threshold, *PHGDH* expression is likely triggered.

The *PHGDH* gene encodes 3-phosphoglycerate dehydrogenase, an enzyme that catalyses 3-phosphoglycerate into 3-phosphohydroxypyruvate in the biosynthesis pathway of L-serine^60^. L-serine is a key enzyme for de-novo sphingolipid synthesis. Sphingolipids are a family of membrane lipids that are involved in diverse functions including lipid metabolism^61^. Sphingolipids can be found in most lipoproteins^62^. Increased intake of dietary sphingolipids have been shown to reduce triglyceride levels in mice^63^ but not in humans^64, 65^. Additionally, the lipid-lowering drug statin has been shown to lower subtypes of plasma sphingolipids in males with metabolomics syndrome^66^. Studies investigating the *PHGDH* gene relationship with sphingolipid levels in humans are needed.

Mutations in the *PHGDH* gene have been associated with phosphoglycerate dehydrogenase deficiency^67^ and Neu Laxova syndrome^68^. No variations in the gene have been associated with triglyceride levels.

The question on the nature of the relationship between blood triglycerides and methylation levels described in this paper is of broad interest: does variation in methylation levels cause variation in triglyceride levels, or vice-versa? In this study, several proximal SNPs associated with methylation levels at cg14476106 were identified. These SNPs can be used as proxies for methylation levels to infer causation through Mendelian randomization approaches^49, 50, 69^. Intuitively, if variation in methylation levels is causing variation in triglyceride levels, then these SNPs should be associated with triglyceride levels. However, there were no strong evidence to show these SNPs being associated with triglyceride levels in our datasets. Additionally, despite the sufficiently large sample size of the Global Lipids Genetics Consortium study, little or marginal effects of SNPs in the neighbourhood of the methylation site were observed on the triglyceride levels (Supplementary Table S3). These results suggest that variation in methylation levels at cg14476106 do not cause variation in fasting triglyceride levels. Thus, it is unlikely that the triglyceride-associated *PHGDH* methylation accounts for the reported triglyceride level missing heritability. However, it is plausible that elevated fasting triglyceride levels decrease methylation levels at this site (reverse causation) promoting the expression of the *PHGDH* gene. Reverse causation can be tested using triglyceride-related SNPs in a Mendelian randomization framework. However, SNP effects on triglyceride levels variation are known to be small, and the datasets available for this study were too small to detect such effects. In a tentative to increase statistical power, a genetic score was developed and tested for association with methylation levels at cg14476106. The genetic risk score explained only a small proportion of triglyceride level variation, which leaves the main findings remain the same. Thus, suggesting that the study datasets were still underpowered or that association between the genetic risk score and methylation levels at cg14476106 is truly absent. Well-powered studies are needed to unequivocally exclude either possibility and to address potential violation of the underlying Mendelian Randomization assumptions.

BMI, a proximate measure for adiposity, is moderately correlated with fasting triglyceride levels. The association between fasting triglycerides and methylation levels may be confounded by BMI. Recently, an association between BMI and methylation levels at cg14476101 was reported^46^. However, triglyceride levels were not considered in that study. In the present study, evidence for association between BMI and methylation levels at cg14476101 was weak. Instead, the results suggested that blood triglycerides lay on the pathway between BMI and *PHGDH* methylation. Thus, the previously reported *PHGDH* methylation associations with BMI may reflect the association with triglycerides.

Using a standard linear model, a difference in effect sizes for the *PHGDH* cg14476101-triglyceride relationship was observed between the discovery and replication datasets. Triglyceride levels, however, were systematically lower in the MARTHA study compared to the F5L family study. Follow-up analyses showed that the differential effect estimates could be explained by the non-linear association between triglyceride levels and DNA methylation at cg14476101. When triglyceride levels were below 1.12 mmol/L, the relationship was flat in the MARTHA study, and mildly declining in the F5L family study. Conversely, above this threshold, the association declined dramatically in both datasets. The threshold effect implies that methylation levels may change (i.e. the CpG site is less methylated) when triglyceride levels are above a certain value.

An alternative explanation for the difference in the strength of association between cg14476101 methylation and triglyceride levels in the MARTHA and F5L family studies is confounding by an unknown factor that affects both variables. However, we used the RUV method to capture cellular heterogeneity as well as other potential unmeasured confounders, and performed sensitivity analyses. Modelling for additional potential confounders (e.g. lipid lowering medication, oral contraceptive use and current smoking) and for SNPs in the vicinity of cg14476101 did not alter the association between triglyceride levels and methylation levels at cg14476101. Thus, the observed difference between the two studies is unlikely due to the above factors.

Other studies have investigated epigenetic variation with triglyceride levels. In a candidate gene study, pyrosequencing of selected genomic targets revealed associations with CpG sites mapped to *ABCG1* and *LIPC* genes in familial hypercholesterolemia^24^. In a genome-wide study using the Infinium HumanMethylation450 BeadChip array in CD4^+^T cells, an association with CpG sites in the *CPT1A* gene were detected^19^, and this association was robustly replicated in independent datasets, including ours^12, 20^. Using the same array on whole blood, another group found associations with CpG sites in *ABCG1* and *SREBF1* genes^25^. Those associations were confirmed in a recent epigenome-wide study between lipid profiles and DNA methylation^23^. The CpG sites in the *SREBF1* gene did not meet the genome-wide significance in our data. The reported strength of the association was small (β = 0.043), suggesting that we likely had insufficient statistical power to detect such small effects (Supplementary Fig. S8). But the statistical power was enough to detect the associations with *ABCG1* and *PHGDH* methylation sites. In addition, the F5L family and MARTHA projects were designed to study, respectively, haemostatic proteins and venous thromboembolism. The ascertainment scheme of these studies may have impacted the observed associations with methylation levels. In particular, methylation levels in the MARTHA study were measured only in patients who developed venous thromboembolism. Nevertheless, these study samples identified the previously reported triglycerides-methylation associations in the *ABCG1* and *CPT1A* genes, supporting the appropriateness of these datasets for epigenetic analyses of lipid-related phenotypes. Recently, a Mendelian Randomization analysis^21^ on 3396 individuals from six studies was performed to investigate the causal relationship between lipid profiles and DNA methylation levels. Results suggest that variations in methylation levels at *CPT1A* and *ABCG1* were a consequence of inter-individual variation of triglyceride levels. Finally, methylation levels at the *TXNIP*, *SLC7A11*, and *MYLIP* were also associated with triglyceride levels^22^. Both the studies were also based on the Infinium HumanMethylation450 BeadChip array.

No association with *PHGDH* DNA methylation was reported in previous triglyceride methylome studies^19, 20, 22, 23, 25^. However, association of *PHGDH* DNA methylation with triglyceride levels was mentioned - but was not followed up - in the Mendelian Randomization study^21^. The threshold effect we observed for the *PHGDH* DNA methylation can affect the effect size estimate, and reduce statistical power in a linear model. Depending on the triglyceride level distribution, the linear model may not have been adequate to detect this association in previous methylome studies.

In summary, a novel association linking increased blood methylation levels at the *PHGDH* cg14476106 with decreased fasting triglyceride levels was discovered. This association may be non-linear with a clinically relevant threshold of approximately 1.12 mmol/L. These results suggest that the decline in methylation levels at cg14476101 may be a consequence, as opposed to a cause, of high triglyceride levels. These findings may bring new insights into the molecular mechanisms underlying triglyceride level variation.

## Electronic supplementary material

Supplementary material
