# Supplementary material for: Blood triglyceride levels are associated with DNA methylation at the serine metabolism gene PHGDH
Source: Sci Rep. 2017 Sep 11;7:11207. doi: 10.1038/s41598-017-09552-z (PMC5593822; doi:10.1038/s41598-017-09552-z)
Supplement: Supplementary file 1 — Supplementary material [file 41598_2017_9552_MOESM1_ESM.pdf]

Blood triglyceride levels are associated with DNA methylation at the serine metabolism gene *PHGDH*

Vinh Truong[1], Siying Huang[1], Jessica Dennis[1], Mathieu Lemire[2], Nora Zwingerman[1], Dylan Aïssi[3,4], Irfahan Kassam[1], Claire Perret[3,4], Philip Wells[5], Pierre-Emmanuel Morange[6], Michael Wilson[7], David-Alexandre Trégouët[3,4], France Gagnon[1*]

[1]Division of Epidemiology, Dalla Lana School of Public Health, University of Toronto, Toronto, Canada

[2]Ontario Institute for Cancer Research, Toronto, Canada

[3] Sorbonne Universités, UPMC Univ. Paris 06, INSERM, UMR_S 1166, Team Genomics & Pathophysiology of Cardiovascular Diseases, Paris, France

[4] ICAN Institute for Cardiometabolism and Nutrition, Paris, France

[5]Department of Medicine, Faculty of Medicine, University of Ottawa, Ottawa, Canada

[6]INSERM, UMR_S 1062, Nutrition Obesity and Risk of Thrombosis, Marseille, France

[7]Genetics and Genome Biology Program, SickKids Research Institute, Toronto, Canada

SUPPLEMENTARY MATERIALS

Technical validation of DNA methylation findings

Technical validation of DNA methylation at cg14476101 by relative quantitative PCR with specific TaqMan probes was performed according to standard protocols. Briefly, sodium bisulfite conversion was performed from 300 ng genomic DNA using EZ-96 DNA Methylation kit (ZymoResearch). From the resulting bisulfite DNA, a relative quantitative PCR was performed using 2 TaqMan MGB probes (Life Technologies) specific to the methylated or unmethylated studied CpG and specific labelled with FAM or VIC reporter. The primers and probes design were performed with Primer Express software from the bisulfite converted DNA sequence. The primers and probes were chosen with no CpG island in their sequence, except the one being studied. The sequence of the probes and primers were: probe C (labelled with FAM) 5'AAGATTTACGTTAGTA3' – probe T (labelled with VIC) 5'AGATTTATGTTAGTAGA3' – primer F 5'TTGAAGGTAGGGATTAAATT3' – primer R 5'CTATCCACCACCTTCAAC3. Relative quantitative PCR was performed on a QuantStudio 3 instrument (Life Technology) with Rox Passive Reference from 1.5µl of bisulfite converted DNA in total volume of 14µl. The reaction mix contained 200 nM of each MGB probe (Life technologies), 900 nM of each primer (Sigma – HPLC purified), 0.1 mM dNTP, 5 mM MgCl2, 0.1X Rox (Euromedex), 0.4U Immolase Taq and 1X ImmoBuffer (Bioline). The amplification program was: 95°C 10 min following by 45 cycles of 95°C 15sec - 56°C 1min. For each sample, the Ct for the two probes were determined with the same threshold, this threshold being defined with control DNA for which methylated value was known. For each sample, the formula "% methylation = $100/[1+2^{(Ct_{meth}-Ct_{unmeth})}]$" was applied to calculate the methylated percentage of the studied CpG.

Results are shown in Supplementary Figure S3.

Genotyping

Genome wide genotypes in the F5L family study were typed using the Illumina 660W-Quad Beadchip. A total of 547,886 autosomal single nucleotide polymorphisms (SNPs) were genotyped in the 255 study subjects. For quality control, SNPs with genotyping call rate ≤ 90% and minor allele count <20 were excluded.

The family structure was verified using 1,079 microsatellite markers and RELPAIR[1] and further verification was completed using PREST[2] to improve the quality control of the genetic data. We examined Mendelian errors in SNPs based on verified family structure information. A total of 14,949 Mendelian errors were identified (error rate = 0.012%). Genotypes showing Mendelian errors were coded as missing. In total, 490,083 autosomal SNPs passed the quality control criteria. The overall genotyping call rate on those SNPs was 99.88%. The average missing rate was 0.13%.

In MARTHA study, 1,592 individuals were first genotyped using the Illumina 610 or 660W-Quad Beadchips. Individuals with genotyping call rate < 95% (n=18) were excluded from the final dataset. To detect individuals having cryptic relatedness, a pairwise clustering of identity-by-state distance and multi-dimensional scaling were performed using the PLINK software[3]. Individuals of non-European ancestry were detected with the Eigenstrat program[4]. SNPs of which genotype frequency deviated from Hardy-Weinberg Equilibrium (HWE) in controls (genome-wide significance, P-value$<10^{-5}$), and with minor allele frequency (MAF) less than 1% or genotyping call rate <99% were filtered out from final dataset. After quality control procedure, a total of 481,002 autosomal SNPs were included in the final dataset.

Statistical model

DNA methylation levels at a CpG site (M-value) were analyzed as outcome and triglyceride levels (log transformed) as a predictor. We used a variance components models in the F5L family study to adjust for the relatedness among family members and the linear model in the MARTHA study.

Since DNA methylation levels can show substantial variation across different cell types[5], the cellular heterogeneity can be a confounding factor (or a mediator) in the epigenetic analysis[6]. However, up to our knowledge, the triglyceride levels are unlikely to be associated with the cell type proportions. In other

hand, the cellular heterogeneity can introduce noise in the analysis and may induce a dependency between the methylation levels (and therefore between the statistical tests). For those reasons, the models should be adjusted for the cell type proportions.

However, the cell type proportions were not measured in the F5L family study. We used the Remove Unwanted Variation (RUV) approach [6-9] to capture the cellular heterogeneity in order to account for their effects on the associations In addition, RUV is able to capture other unwanted variation from the methylation data. Briefly, the method performs a factor analysis on the methylation levels measured at a subset of CpG sites (control probes). The factor components are then included in the regression model to adjust for the unwanted variations. For control probes, we used 473 CpG sites that have been found to be specific to different types of white blood cells[6,10] and whose methylation levels were not associated with log(triglyceride) in the discovery dataset (P-value > 0.10). The RUV components were estimated with the ruv4 method[8] in the *ruv* R-package. We selected the number of components based on the P-value distribution as suggested in Gagnon-Bartsch et al[7].

Finally, the models in the F5L family study were adjusted for age, sex and the three first RUV components. In the MARTHA study, the models were adjusted for age, sex and the proportions of lymphocytes, monocytes, eosinophils and basophils. In the F5L family study, the degrees of freedom in the F-statistics were estimated with the Kenward-Roger approach as implemented in the pkbrtest R-package[11].

Piece-wise linear regression

A segmented relationship between mean response μ=E[Y] and the variable X (with a breakpoint at θ) was modeling by:

$$E[Y] = \begin{cases} \beta_1 X + a & \text{if } X < \theta \\ \beta_2 X + b & \text{otherwise} \end{cases}$$

The model assumed a continuous relationship at X= θ, i.e.

$$\beta_1 \theta + a = \beta_2 \theta + b$$

Instead of constraining the parameters, the model can be reparameterized as:

$$E[Y] = \beta_1 Z_1 + \beta_2 Z_2 + c \quad \text{with} \quad Z_1 = \begin{cases} \theta \ if \ X \geq \theta \\ X \text{ otherwise} \end{cases} \text{ and } Z_2 = \begin{cases} X - \theta \ if \ X \geq \theta \\ 0 \text{ otherwise} \end{cases}$$

According to this parameterization, β_1 is the left slope (slope when $X < \theta$) and β_2 is the right slope (slope when $X > \theta$) and the segments are connected at $Z = \theta$. A Wald test or log-likelihood ratio test can be used to assess if the slopes (β_1 and β_2) are significantly different from 0. In our analyses, Y represents the methylation level at the *PHGDH* CpG site, X represents the log(triglyceride) and θ is the breakpoint (i.e $\theta = \log(1.12)$). Therefore, β_1 is the effect size for triglyceride levels is below 1.12 mmol/L and β_2 are the effect size when triglyceride levels are above 1.1 mmol/L.

Adjustment of cell type heterogeneity

We first evaluated the association of the true cell type proportions of lymphocytes, monocytes and granulocytes on triglyceride levels in the MARTHA study (Supplementary Table S7). Triglyceride levels are associated with monocyte proportion: $\beta = -5.3$, P-value = 0.0017, 95% CI = (-8.5, -2.00). These results suggest that cell type proportions represent a potential confounder of the direct effect of triglyceride levels on the methylation levels at cg14476101.

We then evaluated the quality of the cell type proportion estimations by comparing the true and the estimated cell type proportions in the MARTHA study (Supplementary Table S8, Supplementary Fig S9, S10 and S11). The estimated and true cell type proportions are highly correlated, supporting that the deconvolution method works well on whole blood.

We then report the strength of association of triglyceride levels on the methylation levels at cg14476101 using different adjustments for cell type proportions (Supplementary Table S9) in the MARTHA study. The strength of associations adjusted for the estimated or true cell type proportions are similar. Thus, these results validate our approach to account for cellular heterogeneity in our investigation of the association between blood DNA methylation and triglyceride levels.

Finally, we report the strength of association between triglyceride levels and the methylation levels at cg14476101 adjusting for the estimated cell type proportions in the F5L family study (Supplementary

Table S10). The strength of association estimated from the model adjusted for the RUV components is very similar to the one estimated from the model adjusted for estimated cell type proportions.

Reverse causality

The reverse causality (Figure 1C) was assessed using a weight genetic score. The score was generated with the SNPs reported associated with triglyceride levels in the Global Lipids Genetics Consortium meta-analysis[12]. We excluded two SNPs (rs10761731 and rs11649653) due to the missing information on the strand. The SNPs were weighted using the effect sizes estimated in the meta-analysis. In total, 32 SNPs were included in the genetic score. The detailed information of the 32 SNPs can be found in Table 1 in (Johansen et al, 2011) [13]. All the models were adjusted for age and sex.

We first tested the association between the genetic score and the triglyceride levels (Supplementary Table S4). We observed a significant association in the F5L family Study ($\beta = 0.40$, *P*-value = 0.045) and in the MARTHA study ($\beta = 0.39$, *P*-value=8.7×10^{-4}). However, the evidence of association is not strong in the F5L family study, indicating that our discovery dataset may be underpowered to detect an association between the methylation and the triglyceride levels.

We then evaluated the association of the genetic score on the methylation levels at cg14476101 (Supplementary Table S4). Weak evidence for association was observed in MARTHA ($\beta = -0.13$, *P*-value = 0.11), but not in the F5L Family study ($\beta = -0.089$, *P*-value = 0.48). The sign and size of the estimates in both the studies were consistent with those observed for the association with triglyceride levels. The direction of association between the normalized triglyceride levels and the genetic risk score in a recent study[14] was also consistent with our results ($\beta = -0.8$, 95%CI = (-1.7, 0.1), *P*-value = 0.092). Taken together, these results do not exclude the possibility of an association between the genetic score and methylation levels, but cannot unambiguously support it either. Well-powered studies are needed to answer whether the genetic score is associated with the *PHGDH* CpG methylation levels.

Estimation of the variance explained in the family study

We used a pseudo-R2 [15] to estimate the variance explained by triglyceride levels in the F5L family study. It represents the variance explained by the fixed factors. In our model, we used the following formula:

$$R^2 = \frac{\sigma_f^2}{\sigma_f^2 + \sigma_F^2 + \sigma_e^2}$$

Where σ_f^2 is the variance of the fixed effect components; σ_F^2 and σ_e^2 are the variance components of the model.

REFERENCE

1 Epstein, M. P., Duren, W. L. & Boehnke, M. Improved inference of relationship for pairs of individuals. *American Journal of Human Genetics* **67**, 1219-1231; (2000).

2 Sun, L., Wilder, K. & McPeek, M. S. Enhanced pedigree error detection. *Human Heredity* **54**, 99-110; (2002).

3 Purcell, S. *et al.* Plink: A tool set for whole-genome association and population-based linkage analyses. *American Journal of Human Genetics* **81**, 559-575; (2007).

4 Price, A. L. *et al.* Principal components analysis corrects for stratification in genome-wide association studies. *Nat Genet* **38**, 904-909; (2006).

5 Houseman, E. *et al.* DNA methylation arrays as surrogate measures of cell mixture distribution. *BMC Bioinformatics* **13**, 86; (2012).

6 Jaffe, A. & Irizarry, R. Accounting for cellular heterogeneity is critical in epigenome-wide association studies. *Genome Biology* **15**, R31; (2014).

7 Gagnon-Bartsch, J. A. & Speed, T. P. Using control genes to correct for unwanted variation in microarray data. *Biostatistics* **13**, 539-552; 10.1093/biostatistics/kxr034 (2012).

8 Gagnon-Bartsch, J. A. a. S., T.P. in *Technical Report* (2013).

9 Maksimovic, J., Gagnon-Bartsch, J. A., Speed, T. P. & Oshlack, A. Removing unwanted variation in a differential methylation analysis of illumina humanmethylation450 array data. *Nucleic Acids Research* **43**, e106-e106; (2015).

10 Reinius, L. E. *et al.* Differential DNA methylation in purified human blood cells: Implications for cell lineage and studies on disease susceptibility. *PLoS ONE* **7**, e41361; 10.1371/journal.pone.0041361 (2012).

11 Halekoh, U. & Højsgaard, S. A kenward-roger approximation and parametric bootstrap methods for tests in linear mixed models – the r package pbkrtest. *Journal of Statistical Software; Vol 1, Issue 9 (2014)*; 10.18637/jss.v059.i09 (2014).

12 Willer, C. J. *et al.* Discovery and refinement of loci associated with lipid levels. *Nature genetics* **45**, 10.1038/ng.2797; 10.1038/ng.2797 (2013).

13 Johansen, C. T., Kathiresan, S. & Hegele, R. A. Genetic determinants of plasma triglycerides. *Journal of Lipid Research* **52**, 189-206; 10.1194/jlr.R009720 (2011).

14 Dekkers, K. F. *et al.* Blood lipids influence DNA methylation in circulating cells. *Genome Biology* **17**, 138; 10.1186/s13059-016-1000-6 (2016).

15 Nakagawa, S. & Schielzeth, H. A general and simple method for obtaining r2 from generalized linear mixed-effects models. *Methods in Ecology and Evolution* **4**, 133-142; 10.1111/j.2041-210x.2012.00261.x (2013).

LEGEND FIGURES

Supplementary Figure S1 Manhattan plot showing the distribution of the *P*-values from the methylome association analysis of triglyceride levels in the F5L family study. Associations were tested using a variance components model in the F5L family study where the methylation levels expressed as M-value were analyzed as the outcome, and triglyceride levels as a predictor. Models were adjusted for sex, age and RUV components. Genome-wide significance was assessed with the false discovery rate (q-value ⩽ 0.05). Significant sites are located above the dotted line

Supplementary Figure S2 Quantile-quantile plot of the *P*-values from the methylome association analysis of triglyceride levels in the F5L family study

Supplementary Figure S3 Plot of DNA methylation levels by H450M vs Bisulfite qRT-PCR in the MARTHA study

Supplementary Figure S4 Regional plot of the association of triglyceride levels on the DNA methylation levels in the vicinity of cg14476101 in F5L family Study(circle) and MARTHA (diamond) studies. Blue and pink colors represent negative association respectively in F5L family and in MARTHA. Red and green colors represent positive association.

Supplementary Figure S5 Triglycerides-CpG relationship with BMI. We first estimated the effect of BMI on the methylation levels at the CpG site cg14476101 (a). We evaluated then the impact of adjusting for triglyceride levels on the strength of association between BMI and the methylation levels (b). Conversely, we estimated the impact of adjusting for BMI on the strength of association between triglyceride levels and the methylation levels at cg14476101 (c). Models were adjusted for sex, age and cell type proportions.

Supplementary Figure S6 Path diagram for the mediated effect of BMI on the methylation levels at cg14476101 CpG site through triglyceride levels

Supplementary Figure S7 Annotation of the region of the CpG site cg14476101 with the Roadmap Epigenome browser

Supplementary Figure S8 Strength of the association (95%CI) of triglyceride levels on methylation levels at the *SREBF1* CpG site cg20544516 in the F5L family, MARTHA, KORA F4, KORA F3 and InCHIANTI studies.

Supplementary Figure S9 Plot of measured vs. predicted proportions of lymphocytes in MARTHA study

Supplementary Figure S10 Plot of measured vs. predicted proportions of granulocytes in MARTHA study

Supplementary Figure S11 Plot of measured vs. predicted proportions of monocytes in MARTHA study

SUPPLEMENTARY TABLES

Supplementary Table S1 Effect of triglyceride levels on methylation levels at *PHGDH* cg14476101 without and with adjustment for potential confounders in the F5L family study and in the MARTHA study

	**F5L family study**<br>**Effect size of triglyceride level variation**<br>**(95% CI)**	**MARTHA study**<br>**Effect size of triglyceride level variation**<br>**(95% CI)**
**Without additional adjustment**	-0.21<br>(-0.29, -0.13)	-0.082<br>(-0.16, -6.7×10^{-3})
**Additional adjustment for Lipid-lowering medication (yes/no)**	-0.21<br>(-0.29, -0.13)	-0.082<br>(-0.16, -6.0×10^{-3})
**Additional adjustment for Oral contraceptive use (yes/no)**	-0.22<br>(-0.30, -0.14)	-0.084<br>(-0.16, -3.0×10^{-3})
**Additional adjustment for Current smoking (yes/no)**	-0.21<br>(-0.29, -0.13)	-0.082<br>(-0.16, 2.1×10^{-3})

Associations were tested using a linear regression model (a variance components model in the F5L family study) where cg14476101 methylation levels expressed as M-value were analyzed as the outcome and the potential confounder as a predictor. Each models included adjustment for sex, age and cell type proportions (RUV components in the F5L family study). CI, confidence interval

Supplementary Table S2 Statistically significant associations between methylation levels at *PHGDH* cg14476101 and SNPs in the 1Mb window on each side of the CpG site

		F5L family study Discovery dataset			MARTHA study Replication dataset		
**rs (a1/a2)**	**pos**	**β (95% CI)**	***P*-value**	**Fstat**	**β (95% CI)**	***P*-value**	**F-stat**
**rs454510 (A/G)**	120,195,042	-0.14 (-0.20, -0.07)	7.7×10^{-5}	15.5	-0.12 (-0.17, -0.067)	7.3×10^{-6}	23.4
**rs639216 (C/T)**	120,217,414	-0.15 (-0.22, -0.078)	6.3×10^{-5}	15.6	-0.11 (-0.16, -0.062)	8.0×10^{-6}	23.0
**rs592762 (A/G)**	120,247,355	-0.15 (-0.22, -0.078)	6.3×10^{-5}	15.6	-0.10 (-0.15, -0.056)	1.7×10^{-5}	21.1
**rs942835 (T/C)**	120,263,018	0.27 (0.15, 0.39)	1.5×10^{-5}	19.5	0.21 (0.15, 0.27)	1.9×10^{-11}	47.7
**rs517237 (T/C)**	120,284,988	-0.10 (-0.16, -0.043)	9.0×10^{-4}	10.7	-0.093 (-0.14, -0.047)	9.1×10^{-5}	16.7
**rs532208 (G/T)**	120,292,279	-0.11 (-0.17, -0.05)	3.7×10^{-4}	12.3	-0.084 (-0.13, -0.038)	4.2×10^{-4}	13.7

Associations were tested using a linear regression model (a variance components model in the F5L family study) where cg14476101 methylation levels expressed as M-value were analyzed as the outcome, and the SNP as a predictor by assuming an additive genetic effect. Models were adjusted for sex, age and cell type proportions (RUV components in the F5L family study). Statistical significance was assessed with a FDR in the F5L family study (q-value ≤ 0.05) and with a Holm-Bonferroni correction in the MARTHA study ($p\_corrected \leq 0.05$).

rs, reference SNP ID number; a1, reference allele; pos, location of the SNP on chromosome 1 as per the GRCh37 release; CI, confidence interval; F-stat, F-statistic

Supplementary Table S3. Tested associations between triglyceride levels and the SNPs that were associated with methylation levels at *PGHDH* cg14476101 in the F5L family, MARTHA and Global Lipids Genetics Consortium meta-analysis studies.

	**F5L family study Discovery dataset**		**MARTHA study Replication dataset**		**Global Lipids Genetics Consortium**	
**rs (a1/a2)**	**β (95% CI)**	***P*-value**	**β (95% CI)**	***P*-value**	**β (95% CI)**	***P*-value**
**rs454510 (A/G)**	0.081 (-0.038, 0.20)	0.18	-0.02 (-0.093, 0.053)	0.60	0.0031 (-0.0071, 0.013)	0.46
**rs639216 (C/T)**	0.068 (-0.066, 0.20)	0.31	-0.024 (-0.093, 0.044)	0.49	-0.011 (-0.02, -0.0017)	0.03
**rs592762 (A/G)**	0.068 (-0.066, 0.20)	0.31	-0.0046 (-0.071, 0.061)	0.89	-0.0064 (-0.016, -0.0028)	0.27
**rs942835 (T/C)**	-0.013 (-0.22, 0.20)	0.89	-0.017 (-0.11, 0.072)	0.71	-0.01 (-0.023, 0.0029)	0.20
**rs517237 (T/C)**	0.016 (-0.093, 0.12)	0.77	-0.023 (-0.09, 0.044)	0.50	0.0017 (-0.011, 0.0075)	0.86
**rs532208**	0.026	0.62	-0.0028	0.93	0.0023	0.77

**(G/T)**	(-0.08, 0. 13)		(-0.08, 0.07)		(-0.0067, 0.011)	

Associations were tested using a linear regression model (a variance components model in the F5L family study) where cg14476101 methylation levels expressed as M-value were analyzed as the outcome, and the SNP as a predictor by assuming an additive genetic effect. Models were adjusted on age and sex. Triglyceride levels were log-transformed in the F5L family and MARTHA studies, and were normalized with an inverse normal transformation in the Global Lipids Genetics Consortium meta-analysis.

rs, reference SNP ID number; a1, reference allele

Supplementary Table S4. Association between the triglyceride-associated genetic score and methylation levels at *PHGDH* cg14476101 in the F5L family and MARTHA studies.

	**F5L family study N = 199**	**MARTHA study N = 324**
**Effect size of the genetic score on triglyceride levels**	0.40	0.39
**95% CI**	(6.2×10^{-3}, 0.8)	(0.16, 0.63)
***P*-value**	0.045	8.7×10^{-4}
**F stat**	3.9	12.8
**Effect size of the genetic score on methylation levels at cg14476101**	-0.089	-0.13
**95% CI**	(-0.34, 0.16)	(-0.30, 0.031)
***P*-value**	0.48	0.11

Associations were tested using a linear regression model (a variance components model in the F5L family study) where cg14476101 methylation levels expressed as M-value were analyzed as the outcome, and the triglycerides genetic score as a predictor. Models were adjusted for age and sex.

Supplementary Table S5. Associations of methylation levels at *CPT1A* cg00574958 and at *ABCG1* cg06500161 with levels of triglycerides in the F5L family study and in the MARTHA study.

	**F5L family study**		**MARTHA study**	
	**β (95% CI)**	***P*-value**	**β (95% CI)**	***P*-value**
**cg00574958**	-0.13 (-0.21, -0.04)	1.6×10^{-3}	-0.13 (-0.20, -0.07)	2.3×10^{-5}
**cg06500161**	0.11 (0.073, 0.15)	1.5×10^{-7}	0.11 (0.075, 0.15)	1.92×10^{-8}

Associations were tested using a linear regression model (a variance components model in the F5L family study) where cg14476101 methylation levels expressed as M-values were analyzed as the outcome, and level of lipoprotein as a predictor. Models were adjusted for sex, age and cell type proportion. Cell type proportions were estimated with a deconvolution method in the F5L family study. NA, not available; CI, confidence interval

Supplementary Table S6 Effect of triglyceride levels on methylation levels at *PHGDH* cg14476101 without and with adjustment for potential confounders in the F5L family study and in the MARTHA study.

	**F5L family study Effect size of triglyceride level variation (95% CI)**	**MARTHA study Effect size of triglyceride level variation (95% CI)**
**Without additional adjustment**	-0.21 (-0.29, -0.13)	-0.082 ($-0.16, -6.7 \times 10^{-3}$)
**Additional adjustment for Methylation levels at cg06500161**	-0.22 (-0.31, -0.14)	-0.10 ($-0.19, -1.5 \times 10^{-2}$)
**Additional adjustment for Methylation levels at cg00574958**	-0.21 (-0.30, -0.13)	-0.084 ($-0.17, -7.8 \times 10^{-3}$)

Associations were tested using a linear regression model (a variance components model in the F5L family study) where cg14476101 methylation levels expressed as M-value were analyzed as the outcome and the potential confounder as a predictor. Each models included adjustment for sex, age and cell type proportions (RUV components in the F5L family study). CI, confidence interval

Supplementary Table S7 Association of true cell type proportions on triglyceride levels in the MARTHA study. The models were adjusted for age and sex

	**Estimate**	***P*-value**
**Lymphocyte**	-0.039 (-0.70,0.62)	0.91
**Monocytes**	-5.3 (-8.50, -2.00)	0.00017
**Granulocytes**	0.23 (-0.41,0.86)	0.48

Supplementary Table S8 Pearson's product moment correlation between the true and the estimated cell type proportions in MARTHA study

	**Estimate (95%CI)**
**Lymphocyte**	0.86 (0.83,0.89)
**Monocytes**	0.69 (0.63, 0.74)
**Granulocytes**	0.82 (0.79,0.86)

Supplementary Table S9 Associations of triglyceride levels on methylation levels at cg14476101 CpG site using different adjustment for cell type proportions

	**MARTHA study**
**Adjusted for age and sex**	-0.073 (-0.17, -5.9 $x10^{-3}$)
**Adjusted for age, sex and true cell type proportions**	-0.082 (-0.16, -6.7$x10^{-4}$)
**Adjusted for age, sex and estimated cell type proportions**	-0.085 (-0.17, -0.4 $x10^{-3}$)

Supplementary Table S10 Association of triglyceride levels on methylation levels at cg14476101 CpG site using different adjustment for cell type proportions

	**F5L family study**
**Adjusted for age and sex**	-0.23 (-0.32, -0.15)
**Adjusted for age, sex and RUV components**	-0.21 (-0.29, -0.13)
**Adjusted for age, sex and estimated cell type proportions**	-0.21 (-0.29, -0.13)

Supplementary Table S11. Association of methylation levels at *PHGDH* cg14476101 with triglyceride levels by strata in the F5L family and MARTHA studies.

	F5L family study			MARTHA study		
	**N**	**β (95% CI)**	***P*-value**	**N**	**β (95% CI)**	***P*-value**
**triglyceride levels ≥ 1.69 mmol/L**	57	-0.40 (-0.58, -0.22)	1.8×10^{-5}	36	-0.29 (-0.68, 0.11)	0.15
**triglyceride levels < 1.69 mmol/L**	142	-0.10 (-0.23, 0.027)	0.12	288	-0.053 (-0.15, 0.044)	0.28

Associations were tested using a linear regression model (a variance components model in the F5L family study) where cg14476101 methylation levels expressed as M-values were analyzed as the outcome, and triglyceride levels as a predictor. We estimated the effects by triglyceride levels strata (triglyceride levels ≥ 1.69 mmol/L (150 mg/dL) and triglyceride levels < 1.69 mmol/L) using a piecewise linear regression model. Models were adjusted for age, sex and cell type proportion (RUV component in the F5L family study).

CI, confidence interval; N, number of individuals available for analysis

FIGURES

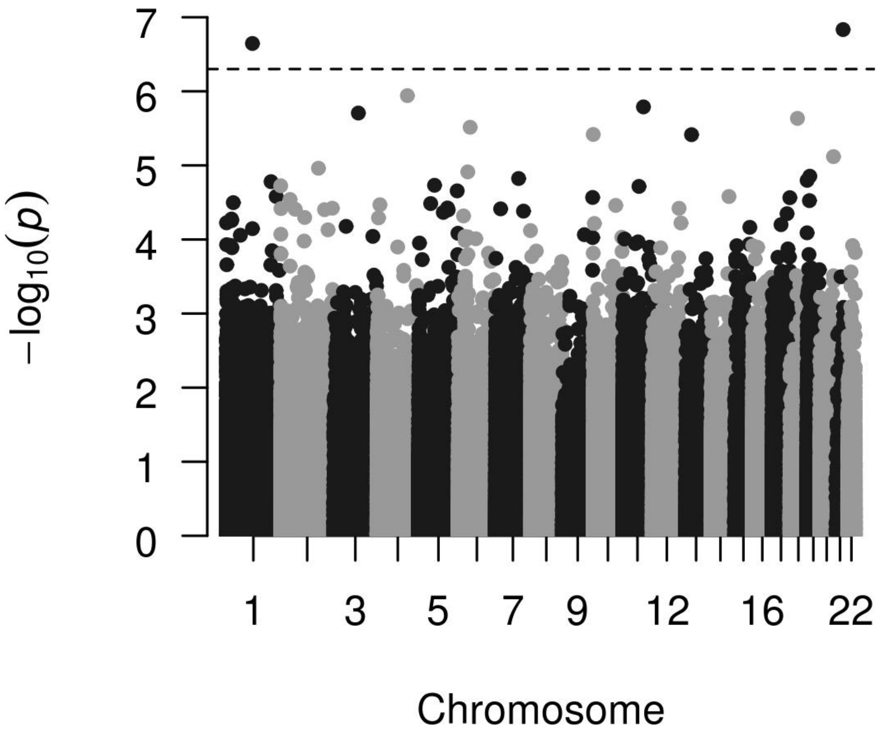

Supplementary Figure S1 Manhattan plot showing the distribution of the *P*-values from the methylome association analysis of triglyceride levels in the F5L family study. Associations were tested using a variance components model in the F5L family study where the methylation levels expressed as M-value were analyzed as the outcome, and triglyceride levels as a predictor. Models were adjusted for sex, age and RUV components. Genome-wide significance was assessed with the false discovery rate (q-value ≤ 0.05). Significant sites are located above the dotted line

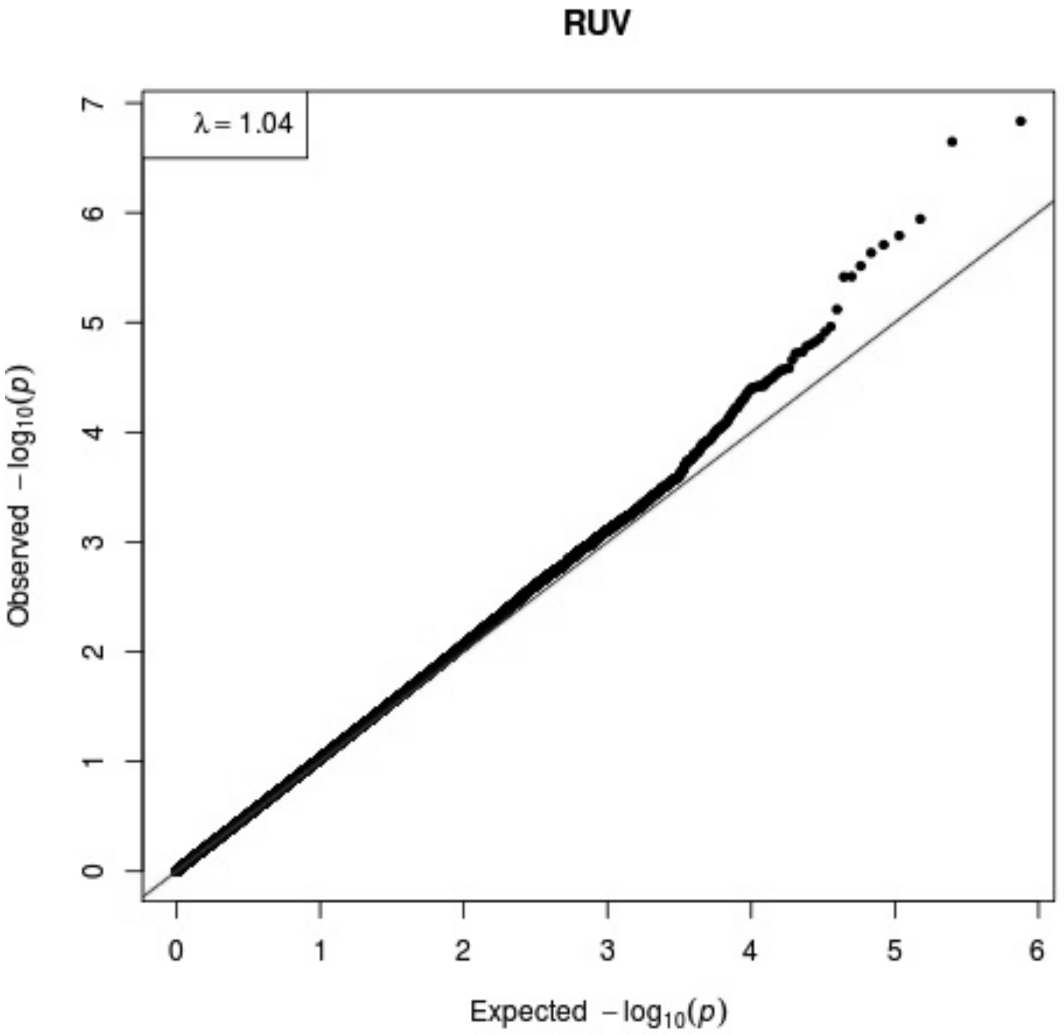

Supplementary Figure S2 Quantile-quantile plot of the *P*-values from the methylome association analysis of triglyceride levels in the F5L family study

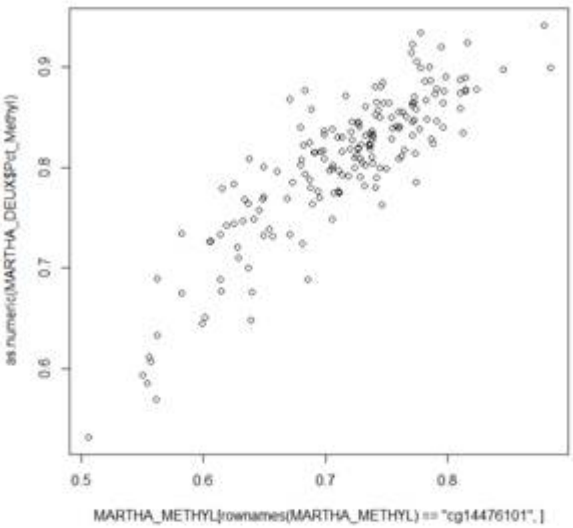

Supplementary Figure S3 Plot of DNA methylation levels by H450M vs Bisulfite qRT-PCR in the MARTHA study

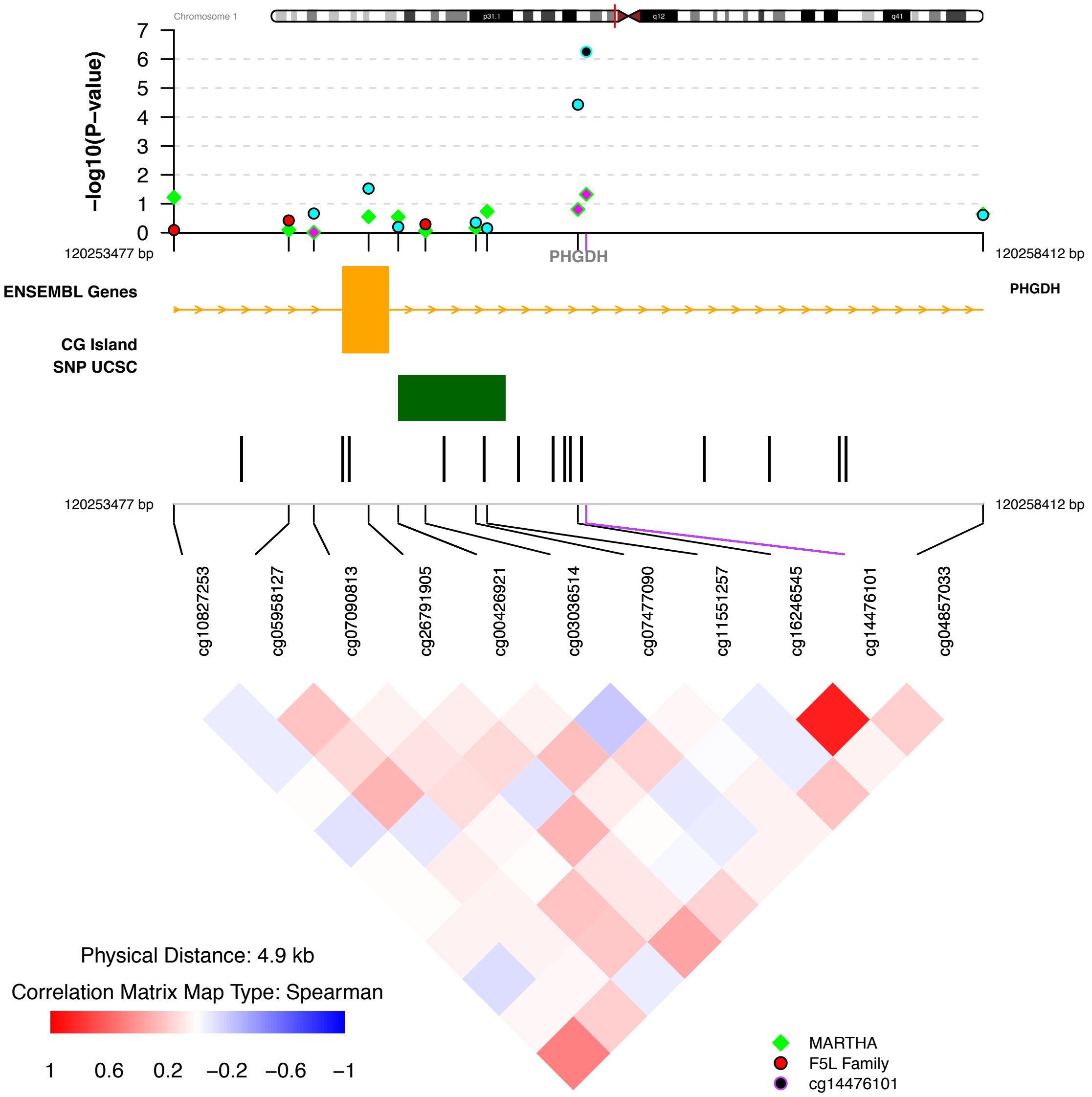

Supplementary Figure S4 Regional plot of the association of triglyceride levels on the DNA methylation levels in the vicinity of cg14476101 in F5L family Study(circle) and MARTHA (diamond) studies. Blue and pink colors represent negative association respectively in F5L family and in MARTHA. Red and green colors represent positive association.

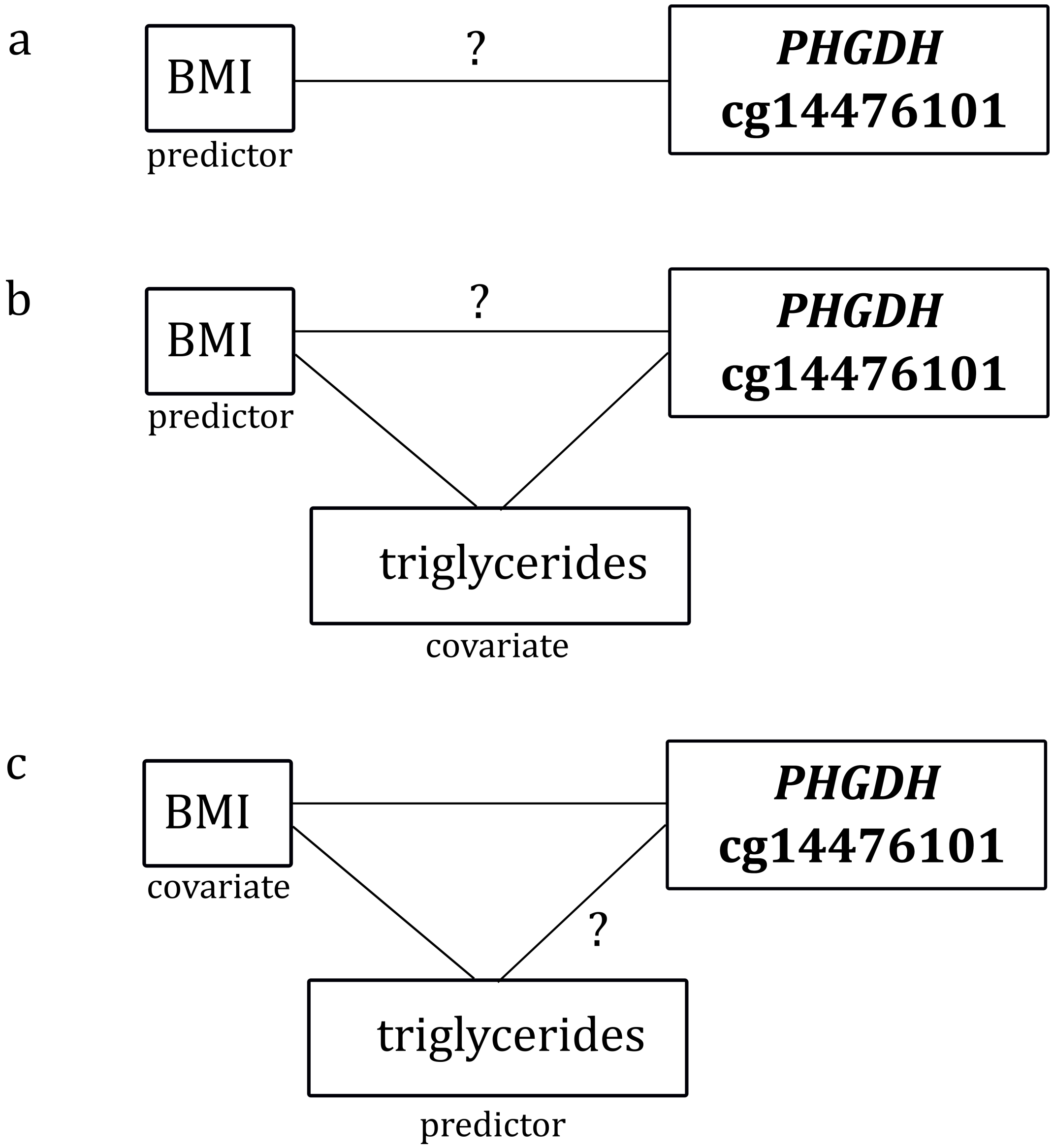

Supplementary Figure S5 Triglycerides-CpG relationship with BMI. We first estimated the effect of BMI on the methylation levels at the CpG site cg14476101 (a). We evaluated then the impact of adjusting for triglyceride levels on the strength of association between BMI and the methylation levels (b). Conversely, we estimated the impact of adjusting for BMI on the strength of association between

triglyceride levels and the methylation levels at cg14476101 (c). Models were adjusted for sex, age and cell type proportions.

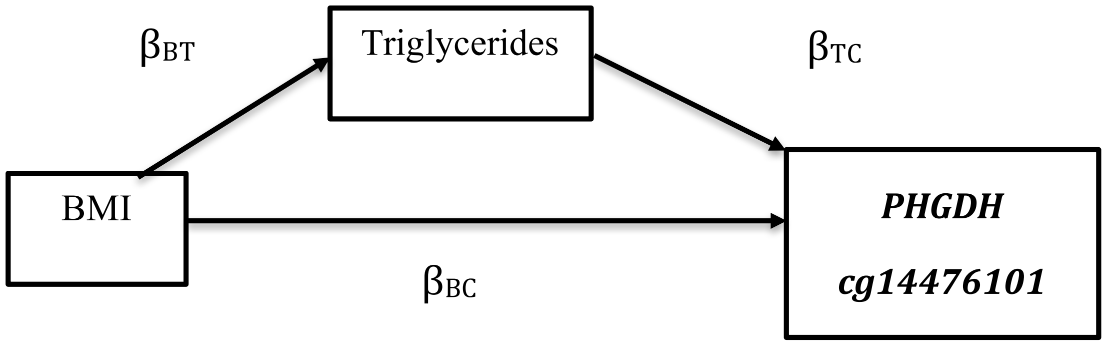

Supplementary Figure S6 Path diagram for the mediated effect of BMI on the methylation levels at cg14476101 CpG site through triglyceride levels

.

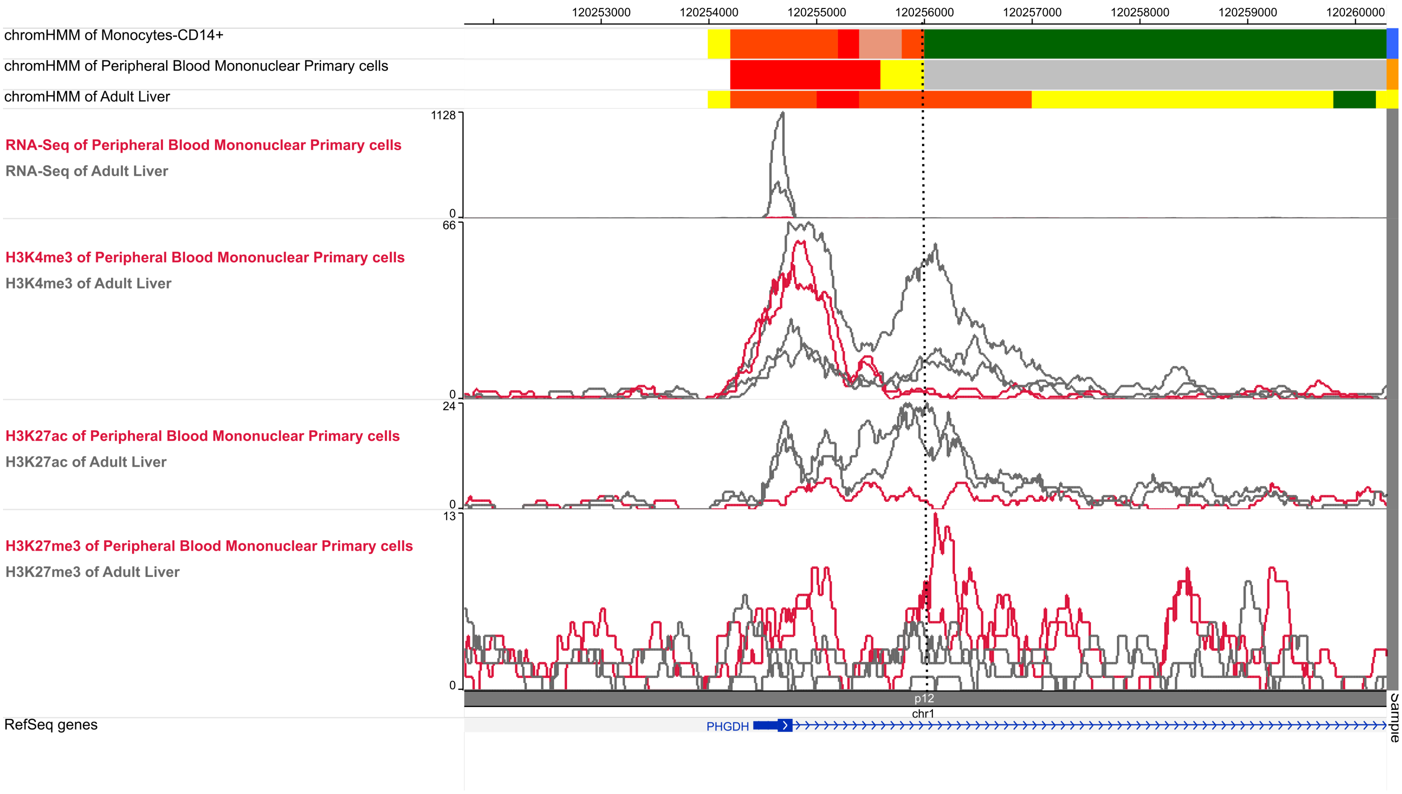

Supplementary Figure S7 Annotation of the region of the CpG site cg14476101 with the Roadmap Epigenome browser

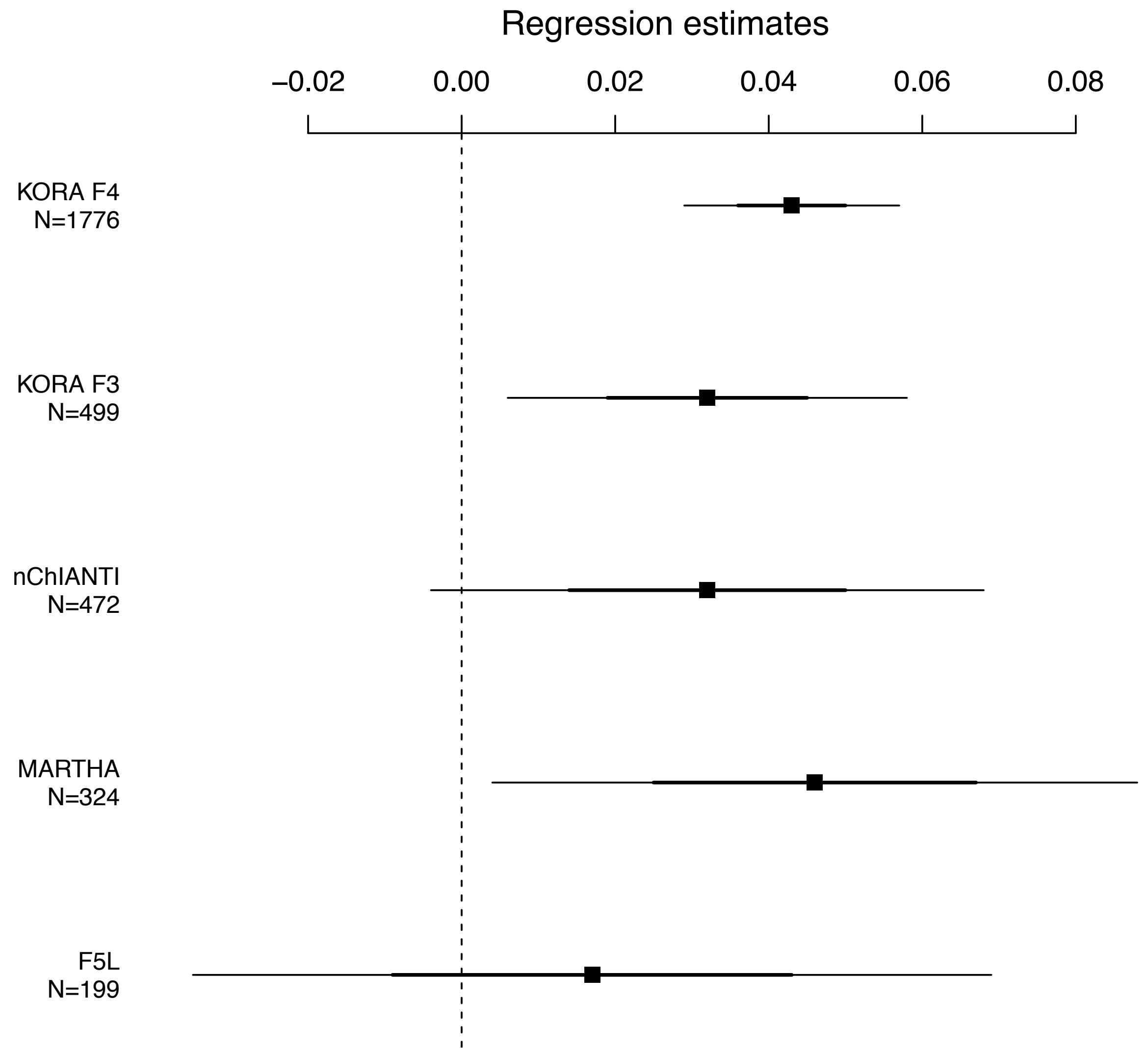

Supplementary Figure S8 Strength of the association (95%CI) of triglyceride levels on methylation levels at the *SREBF1* CpG site cg20544516 in the F5L family, MARTHA, KORA F4, KORA F3 and InCHIANTI studies.

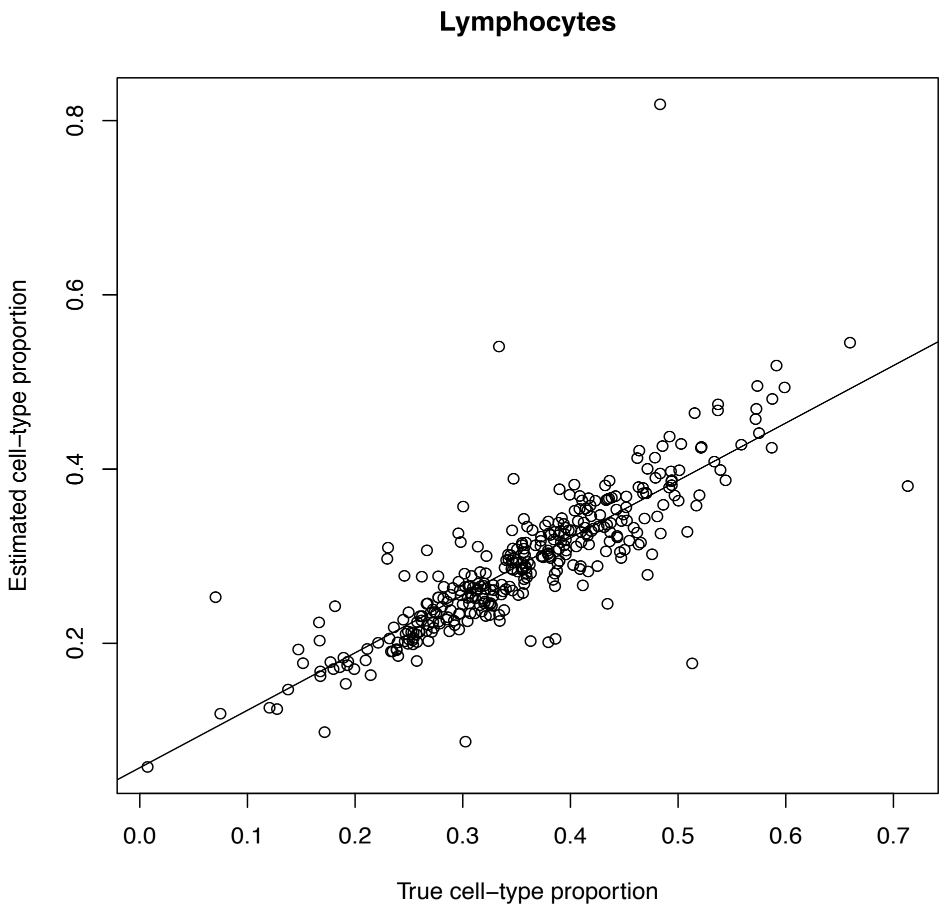

Supplementary Figure S9 Plot of measured vs. predicted proportions of lymphocytes in MARTHA study

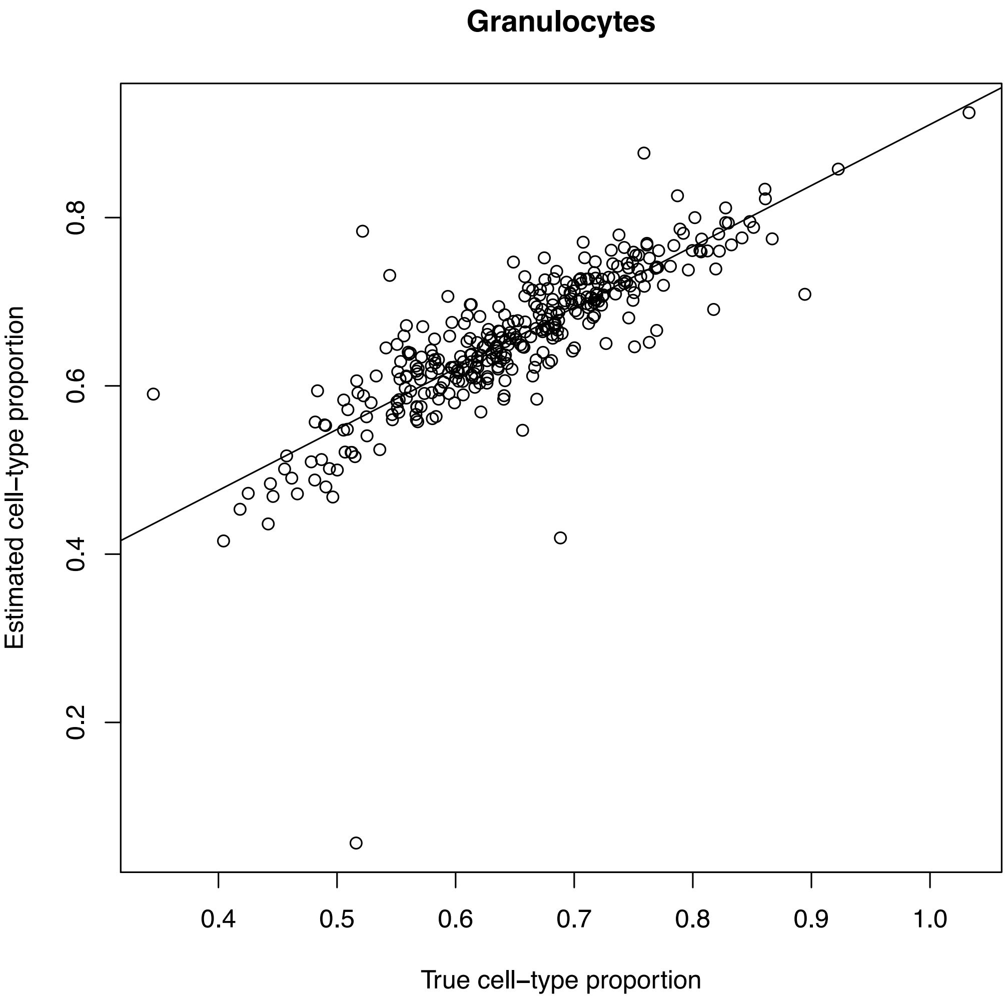

Supplementary Figure S10 Plot of measured vs. predicted proportions of granulocytes in MARTHA study

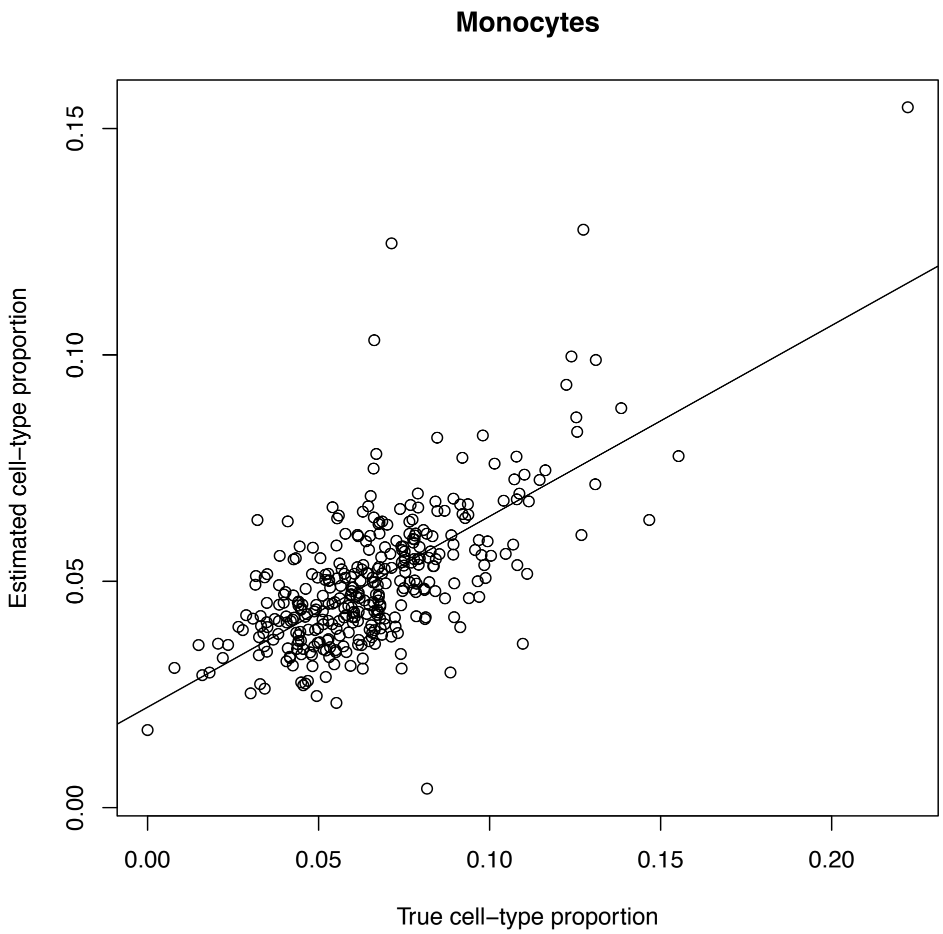

Supplementary Figure S11 Plot of measured vs. predicted proportions of monocytes in MARTHA study
